# An essential Noc3p dimerization cycle mediates ORC double-hexamer formation in replication licensing

**DOI:** 10.26508/lsa.202201594

**Published:** 2023-01-04

**Authors:** Aftab Amin, Rentian Wu, Muhammad Ajmal Khan, Man Hei Cheung, Yanting Liang, Changdong Liu, Guang Zhu, Zhi-Ling Yu, Chun Liang

**Affiliations:** 1 Division of Life Science, Center for Cancer Research, and State Key Lab of Molecular Neuroscience, Hong Kong University of Science and Technology, Hong Kong, China; 2 School of Chinese Medicine, Hong Kong Baptist University, Hong Kong, China; 3 EnKang Pharmaceuticals (Guangzhou), Ltd., Guangzhou, China

## Abstract

Replication licensing, a prerequisite of DNA replication, helps to ensure once-per-cell-cycle genome duplication. Some DNA replication-initiation proteins are sequentially loaded onto replication origins to form pre-replicative complexes (pre-RCs). ORC and Noc3p bind replication origins throughout the cell cycle, providing a platform for pre-RC assembly. We previously reported that cell cycle–dependent ORC dimerization is essential for the chromatin loading of the symmetric MCM double-hexamers. Here, we used *Saccharomyces cerevisiae* separation-of-function *NOC3* mutants to confirm the separable roles of Noc3p in DNA replication and ribosome biogenesis. We also show that an essential and cell cycle–dependent Noc3p dimerization cycle regulates the ORC dimerization cycle. Noc3p dimerizes at the M-to-G_1_ transition and de-dimerizes in S-phase. The Noc3p dimerization cycle coupled with the ORC dimerization cycle enables replication licensing, protects nascent sister replication origins after replication initiation, and prevents re-replication. This study has revealed a new mechanism of replication licensing and elucidated the molecular mechanism of Noc3p as a mediator of ORC dimerization in pre-RC formation.

## Abstract (precis)

Differential mutants confirmed Noc3’s separate roles in DNA replication and ribosome biogenesis, and an essential Noc3 dimerization cycle was found to mediate ORC dimerization in replication licensing.

## Introduction

Faithful genomic duplication is essential for cell viability and survival. Chromosomal DNA replication is stringently regulated to ensure that genome duplication occurs exactly once per cell cycle (Tanaka et al, 1997; Zhai et al, 2010; Diffley, 2011; Zhai et al, 2011; Costa et al, 2013; Amin et al, 2019, 2020; Kyei Barffour & Acheampong, 2021; Lin & Prasanth, 2021). Origins of DNA replication are marked and protected by the origin recognition complex (ORC), which forms a loading pad for the recruitment of various other DNA replication-initiation proteins to form pre-replicative complexes (pre-RCs) at replication origins during the M-to-G_1_ transition (Zhang et al, 2002; Chen et al, 2007; Lee et al, 2010; Ma et al, 2010; Huo et al, 2012; Huang et al, 2016b; Cheung et al, 2019; Wang et al, 2022). The completion of pre-RC formation (also referred to as replication licensing/origin licensing) entails the loading of the head-to-head Mcm2-7p double-hexamer at origins of replication (Evrin et al, 2009; Remus et al, 2009). At the G_1_-to-S transition, after pre-RC formation, Mcm2-7p and other initiation proteins are activated by cyclin-dependent kinases and Dbf4p-dependent kinase (Zegerman & Diffley, 2006; Sheu & Stillman, 2010; Tanaka & Araki, 2010; Heller et al, 2011; Yeeles et al, 2015).

The initiation of DNA replication is monitored by checkpoints to ensure that DNA replication synchronizes with other cellular processes such as protein synthesis, cell growth, mitosis, and cytokinesis. Ribosome biogenesis, in which ribosomal components are synthesized, processed, and assembled to generate new ribosomes, serves as a key marker of the cell’s nutritional conditions and of cell growth (Rudra & Warner, 2004). In actively growing cells, ∼60% of the energy and nutrition are used for ribosome biogenesis (Rudra & Warner, 2004). Ribosome biogenesis takes place in the nucleolus, a dynamic structure composed of ribosomal DNA, rRNA, ribosomal proteins, and ribosome biogenesis proteins. The Noc complex comprising of Noc1p, Noc2p, and Noc3p, with Noc3p primarily forming a heterodimer with Noc2p, is involved in the formation of the 40S ribosome subunits, 60S subunit maturation, and intranuclear transportation of ribosome subunits (Milkereit et al, 2001; Dlakić & Tollervey, 2004; Bassler et al, 2006). Noc3 is also involved in cell differentiation, growth, development, apoptosis, and chromatin re-modeling (Massari & Murre, 2000; Robinson & Lopes, 2000).

It has also been established that Noc3p plays direct roles in budding yeast and human DNA replication, although the molecular mechanism of action remains unclear (Zhang et al, 2002; Tominaga et al, 2004; Johmura et al, 2008a, 2008b; Huo et al, 2012; Cheung et al, 2019).

Several lines of evidence show that DNA replication is regulated by some proteins that also function in ribosome biogenesis and other processes. In addition to Noc3p, the involvement of other ribosome biogenesis proteins such as yeast Ipi3p (Huo et al, 2012; Huang et al, 2016b) and Yph1p (Du & Stillman, 2002) in DNA replication has also been reported. On the contrary, the replication-initiation protein Cdc6p is also involved in ribosomal DNA transcription initiation (Huang et al, 2016a). Moreover, besides the multifunctional Noc3p, Ipi3p, and Cdc6p proteins, other initiation proteins such as ORC and MCM also have roles outside of DNA replication, in processes such as transcriptional regulation (Bueno & Russell, 1992; Foss et al, 1993; Loo et al, 1995; Yankulov et al, 1999; Calzada et al, 2001; Weinreich et al, 2001).

In addition to its role in pre-RC formation during the M-to-G_1_ transition, the chromatin association of Noc3p stabilizes pre-RCs in G_1_-phase (Zhang et al, 2002; Cheung et al, 2019). Noc3p, like ORC, is also continuously bound to chromatin throughout the cell cycle (Zhang et al, 2002; Amin et al, 2019; Cheung et al, 2019; Amin et al, 2020). Noc3p directly interacts with ORC and other initiation proteins such as Ipi3p, Cdc6p, and MCM at replication origins to form and maintain pre-RCs (Zhang et al, 2002; Huo et al, 2012; Huang et al, 2016b; Cheung et al, 2019; Wu et al, 2019).

Although many initiation proteins have been identified, the mechanisms of their actions and of pre-RC assembly remain unclear. Several cryo-electron microscopy (cryo-EM) and single-molecule studies using purified proteins have aimed to elucidate the structures of pre-RC components to discern the mechanisms of replication licensing. The “one-ORC” model, based primarily on in vitro studies, proposes that the architecture of ORC1-6p is asymmetrical, and a single ORC hexamer recruits a single Cdt1p-Mcm2-7p complex, which subsequently recruits a second Cdt1p-Mcm2-7p complex (Sun et al, 2012, 2013, 2014; Li et al, 2015; Ticau et al, 2015; Coster & Diffley, 2017; Yuan et al, 2017; Zhai et al, 2017; Miller et al, 2019). The “two-ORC” model states that two ORC single-hexamers, each bound to one origin, simultaneously and synergistically recruit Mcm2-7p at a >sevenfold higher efficiency than an ORC single-hexamer (Coster & Diffley, 2017).

A cryo-EM study reported that 70% of the ORC-bound origin DNA had one ORC (Orc1-6p) at the ACS, 4% had one ORC at the B2 element, and 26% had two ORCs (one at the ACS and another at the B2 element) in either an inverted (18%) or a tandem (8%) orientation, and that eight different patterns of ORC- and MCM-bound origins were found, with each origin having one or two MCMs together with one or two ORCs (Miller et al, 2019). It was proposed that two MCM rings are sequentially loaded in a similar way by one or two ORCs at the same origin (Miller et al, 2019). Although the biological significance of two ORCs at the same origin was not elaborated, it was suggested that two ORCs at one origin load two MCMs sequentially, making this model significantly different from both the one-ORC and two-ORC models. In addition to the reports of two ORCs occupying two different sites at one origin (Miller et al, 2019) or two origins (Coster & Diffley, 2017), ORC dimers (stated as 5–10% of the purified ORC) have also been observed, but not elaborated (Sun et al, 2012; Ticau et al, 2015).

Recently, we reported in vivo data to support an “ORC dimerization cycle” model, suggesting that non–chromatin-bound ORC interacts with chromatin-bound ORC to dimerize at origins of replication to enable pre-RC formation at the M-to-G_1_ transition, and de-dimerization occurs in S-phase upon origin firing (Amin et al, 2020). This model suggests that symmetrical ORC dimers provide symmetric platforms for the simultaneous and synergistic loading of symmetric pre-RCs at origins (Amin et al, 2020). A recent single-molecule study has also reported one and two ORCs binding to a single ARS, and the authors proposed that both one- and two-ORC mechanisms may be active in vivo (Gupta et al, 2021).

Significantly, the ORC dimerization cycle model (Amin et al, 2020) proposes that as ORC is continuously bound to the chromatin throughout the cell cycle (Liang & Stillman, 1997; Weinreich et al, 1999) and de-dimerizes in S-phase without a net gain of ORC on chromatin (Amin et al, 2020), both the parental and newly replicated origins at each locus are protected by ORC single-hexamers from potential invasion and subsequent inactivation by histones, which have a high affinity for DNA, particularly at A/T-rich sequences at replication origins (Moyle-Heyrman et al, 2013). Of note, ORC binding sites that have conserved nucleosome depletion patterns are likely to be true origins, further suggesting that ORC association with origins is required for accurate nucleosome configuration and hence proper origin selection and function (Lipford & Bell, 2001; Eaton et al, 2010; Tsai et al, 2014).

Noc3p is continuously bound to the chromatin throughout the cell cycle, probably through its interactions with the four ORC subunits (Orc2p, Orc3p, Orc5p, and Orc6p) that form the lower two-thirds of the Orc1-6p hexamer structure (Zhang et al, 2002; Huo et al, 2012; Wu et al, 2019). Noc3p can self-interact (Wu et al, 2019), similar to Orc1-6p (Huo et al, 2012; Wu et al, 2019; Amin et al, 2020). This raises the possibility that Noc3p may mediate ORC dimerization.

In this report, we show that different separation-of-function yeast mutants of *NOC3* have varying proficiencies and deficiencies in ribosome biogenesis and DNA replication, that Noc3p is required for ORC dimerization and MCM loading at origins of replication, that Noc3p monomers self-interact and dimerize in a cell cycle–regulated manner, that free Noc3p and ORC proteins bind chromatin to form (Noc3p-ORC)_2_ dimers in late M-phase right before MCM loading, and that Noc3p de-dimerizes in S-phase. Our findings uncovered an essential, cell cycle–dependent, and likely semi-conservative “dimerization cycle” of Noc3p that mediates and couples with ORC dimerization to regulate eukaryotic chromosomal DNA replication. This is the first time since the discovery of Noc3p as a novel replication-initiation protein (Zhang et al, 2002) that the molecular mechanism of Noc3p as a mediator of ORC dimerization in pre-RC formation has been elucidated.

## Results

### Noc3p plays essential but separable roles in ribosome biogenesis and DNA replication

Earlier studies reported that Noc3p is involved in the process of ribosome biogenesis (Milkereit et al, 2001). It was later established that Noc3p is also essential for the initiation of DNA replication in budding yeast and human cells (Zhang et al, 2002; Huo et al, 2012; Cheung et al, 2019). To further understand the molecular mechanisms of Noc3p in replication licensing and the multifunctional nature of Noc3p, we generated *NOC3* temperature-sensitive (ts) mutants by random mutagenesis of *NOC3*.

**Figure S1.**
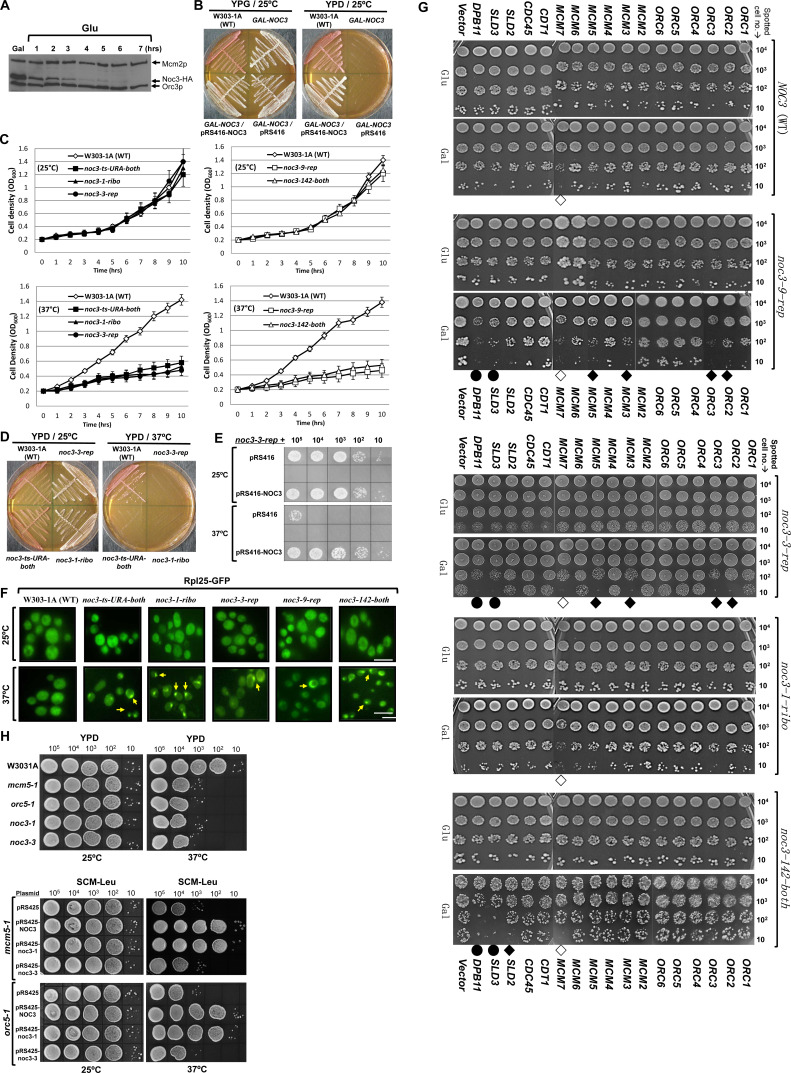
Additional data together with Fig 1 to verify the *NOC3* separation-of-function (replication versus ribosome biogenesis) mutants; Noc3p is essential for cell growth and survival; *NOC3* and *noc3-1-ribo*, but not *noc3-3-rep*, are multicopy suppressors of the *mcm5-1* and *orc5-1* mutant cells. **(A)** Noc3p was mostly degraded by 4 h and was no longer detectable by 6 h after the GAL-NOC3 cells were shifted from galactose-containing medium (YPG) to glucose-containing medium (YPD). Mcm2p and Orc3p were used for comparison. **(B)** W303-1A, GAL-NOC3, GAL-NOC3/pRS416, and GAL-NOC3/pRS416-NOC3 cells were streaked on YPD and YPG plates. The plates were incubated at 25°C for 3–5 d. **(C)** Growth curves of the WT and NOC3 ts mutants when the asynchronous cells were cultured at 25°C or shifted to 37°C. Cell densities were measured at one-hour intervals. Results were the average ± SD of three independent experiments. **(D)** W303-1A, noc3-3-rep, noc3-1-ribo, and noc3-ts-URA-both cells were streaked onto YPD plates and incubated at 25°C or 37°C for 3–5 d. **(E)** 10-fold serial dilutions of noc3-3-rep cells containing pRS416 or pRS416-NOC3 plasmid were spotted onto SCM-Ura (synthetic complete medium lacking uracil) plates and grown at 25°C or 37°C for 3–5 d. **(F)** Microscopic images of nuclear-accumulated Rpl25-GFP in G1-phase–synchronized WT cells and those of five different NOC3 mutant strains grown at 25°C and 37°C (n ≥ 300 for each cell sample). Localization was visualized under a fluorescence microscope within 10 min of harvesting. Scale bar, 10 µm. Magnification, 600×. Arrowheads indicate nuclear accumulation. The NOC3 mutants are denoted with a postfix “-rep” (severely defective in DNA replication with mild defects in ribosome biogenesis), “-ribo” (severely defective in ribosome biogenesis without obvious defect in DNA replication), or “-both” (with intermediate levels of deficiencies in both ribosome biogenesis and DNA replication), for easy reference. **(G)** 10-fold serial dilutions of NOC3, noc3-1-ribo, noc3-3-rep, noc3-9-rep, and noc3-142-both cells containing the plasmids overexpressing the indicated initiation proteins were spotted onto plates containing galactose or glucose and grown at 25°C. The open diamonds indicate that the genes induced dominant-negative effects in all strains. The closed diamonds indicate that the genes caused NOC3 mutant allele-specific dosage lethality phenotypes. The closed circles indicate that the genes induced dominant-negative effects in all replication-deficient NOC3 mutant strains. **(H)** 10-fold serial dilutions of the mcm5-1 and orc5-1 mutant cells transformed with the multicopy plasmids pRS425-NOC3, pRS425-noc3-1, or pRS425-noc3-3, or the vector pRS425, were spotted on SCM-Leu (synthetic complete medium lacking leucine) plates and grown at 25°C or 37°C for 4 d. Untransformed strains (upper panels) were used as controls to show the similar temperature sensitivities of the mutants.

We first generated a *GAL*-*NOC3* yeast strain to facilitate *NOC3* mutant selection and analysis by inserting the galactose-inducible promoter to control the endogenous WT *NOC3* gene. After changing from galactose medium to glucose medium for 6 h, Noc3p was depleted to an undetectable level in *GAL*-*NOC3* cells (Fig S1A). Furthermore, the expression of ectopically expressed WT Noc3p (pRS416-NOC3) rescued the cells grown on glucose-containing plates, whereas the empty vector (pRS416) did not (Fig S1B). This strain was then used to reveal the temperature sensitivities of *NOC3* mutants when *GAL*-*NOC3* expression is suppressed.

After *NOC3* random mutagenesis, several *NOC3* ts mutants were identified and integrated into the W303-1A host cells, replacing the endogenous *NOC3*. Three such mutants, *noc3-3*, *noc3-9*, and *noc3-142*, were characterized in detail in this study. Two published *NOC3* ts mutants from others, *noc3-1* (Milkereit et al, 2001) and *noc3-ts-URA* (Ben-Aroya et al, 2008), and the WT control (W303-1A) were also included in the study. Of note, because these *NOC3* mutants show differential defects in ribosome biogenesis and DNA replication (see below), we named these separation-of-function mutants with a postfix “*-rep*” (severely defective in DNA replication with mild defects in ribosome biogenesis), “*-ribo*” (severely defective in ribosome biogenesis without obvious defects in DNA replication), or “*-both*” (with intermediate levels of deficiencies in both ribosome biogenesis and DNA replication), for easy reference.

The cell density of the WT cells at both 25°C and 37°C, and of mutant cells at 25°C increased normally over time (Fig S1C). However, all five *NOC3* mutant strains showed comparably severe growth defects at 37°C, indicating that these mutants have similar temperature sensitivities, which is important when the differential defects in ribosome biogenesis and DNA replication of these mutants were compared under the same condition (see below). On solid plates, WT cells, but not the *noc3-1-ribo*, *noc3-ts-URA-both*, or *noc3-3-rep* cells, could grow at 37°C (Fig S1D). Furthermore, WT *NOC3* (pRS416-NOC3), but not the empty vector (pRS416), could rescue the growth of *noc3-3-rep* cells (Fig S1E). These data verified the ts growth phenotypes of the *NOC3* mutants.

**Figure 1.**
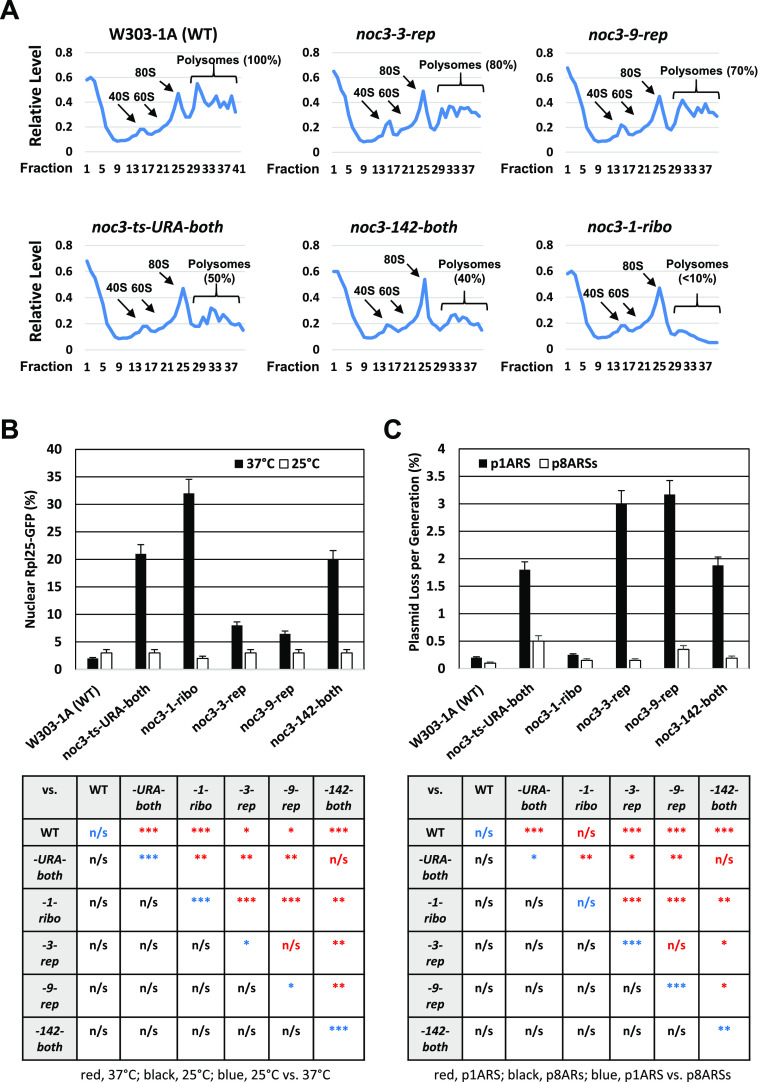
*NOC3* separation-of-function mutants show differential defects in DNA replication initiation and ribosome biogenesis. **(A)** Ribosome profiles (OD_260_) for the WT and five *NOC3* ts strains synchronized in G_1_-phase by α-factor before being shifted to 37°C. Equal amounts of cell lysates were fractionated through 20–60% sucrose gradients. The 40S small subunit, 60S large subunit, and 80S mono-ribosome and poly-ribosome were indicated by arrows. A normal polysome profile is indicated as 100%, whereas other values show different levels of polysome defects. The OD_260_ reading of sucrose gradient fractions was measured using a spectrophotometer. The averages from three independent experiments are shown. **(B)** Quantification of the nuclear-accumulated Rpl25-GFP in WT and six different *NOC3* mutants at 25°C and 37°C (n ≥ 300 for each cell sample). **(C)** Quantitative plasmid loss rates were measured for the WT and six *NOC3* strains containing either p1ARS or p8ARSs grown in YPRG medium at 30°C as indicated. Plasmid loss rates are expressed as the average percentage of loss per generation. Results were the average ± SD of three independent experiments. Statistical analysis was carried out by one-Way ANOVA and Dunnett’s multiple comparison test. Not significant (n/s), *P* > 0.05; *, *P* < 0.05; **, *P* < 0.01; and ***, *P* < 0.001. The *NOC3* mutants are denoted with a postfix “*-rep*” (severely defective in DNA replication with mild defects in ribosome biogenesis), “*-ribo*” (severely defective in ribosome biogenesis without obvious defect in DNA replication), or “*-both*” (with intermediate levels of deficiencies in both ribosome biogenesis and DNA replication), for easy reference.

We then examined ribosome biogenesis and DNA replication phenotypes in different *NOC3* mutant cells. Sucrose gradient sedimentation was performed to examine the ribosome profiles of α-factor synchronized WT (W303-1A) and the *NOC3* mutant cells shifted to 37°C for 3 h (Fig 1A). Contrary to WT cells, which gave a normal poly-ribosome profile, poly-ribosomes were almost completely absent in *noc3-1-ribo* cells, consistent with the reported severe ribosome biogenesis defects (Milkereit et al, 2001). The levels of poly-ribosomes in *noc3-3-rep* and *noc3-9-rep* cells were at about 70–80% of the WT level, indicating mild defects in ribosome biogenesis. The amounts of poly-ribosomes in *noc3-ts-URA-both* and *noc3-142-both* cells were more significantly reduced (at about 40–50% of the WT level), albeit not as significant as in *noc3-1-ribo* cells, indicating moderate defects in ribosome biogenesis.

The different levels of ribosome biogenesis defects observed by sucrose gradient experiments were further verified by Rpl25-GFP localization assays. Rpl25p is a component of the ribosome large subunit. Previous studies reported that Rpl25p mainly localizes in the cytoplasm of WT cells, while accumulating in the nucleus of *noc3-1-ribo* cells (Milkereit et al, 2001). We found that Rpl25-GFP localized in the cytoplasm of the WT and all *NOC3* mutant cells at 25°C and also in WT cells at 37°C (less than ∼4% nuclear accumulation; Figs 1B and S1F). At 37°C, however, the percentages of cells with nuclear Rpl25-GFP accumulation in the mutants increased as follows: *noc3-1-ribo*, 32%; *noc3-ts-URA-both*, 21%; *noc3-142-both*, 20%; *noc3-3-rep*, 9%; and *noc3-9-rep*, 6%. These results show that *noc3-1-ribo* cells have severe defects in ribosome biogenesis, *noc3-ts-URA-both* and *noc3-142-both* cells have moderate defects, and *noc3-3-rep* and *noc3-9-rep* cells have only mild defects. These data are consistent with the sucrose gradient results (Fig 1A).

To examine the efficiency of DNA replication initiation, quantitative plasmid loss assays were performed for the WT and the *NOC3* mutant cells grown at a semi-permissive temperature of 30°C. Replication-initiation mutants are known to have high loss rates for the p1ARS plasmid containing a single *ARS1*, but lower loss rates for p8ARSs, which carries additional tandem copies of the *H4-ARS* (Zhang et al, 2002; Ma et al, 2010; Huo et al, 2012).

As shown in Fig 1C, the p8ARS loss rates were relatively low in the WT and all of the mutant strains (0.1–0.35%). However, the p1ARS plasmid loss rates in the *–rep* or *–both* mutants, but not *noc3-1-ribo*, increased, as follows: *noc3-9-rep*, 3.2%; *noc3-3-rep*, 3.0%; *noc3-142-both*, 1.9%; *noc3-ts-URA-both*, 1.8%; *noc3-1-ribo*, 0.25%; and W303-1A, 0.20%. These results show that the ribosome biogenesis–proficient *noc3-9-rep* and *noc3-3-rep* cells (Figs 1A and B and S1F) have severe defects in DNA replication initiation, whereas *noc3-142-both* and *noc3-ts-URA-both* cells, which are moderately defective in ribosome biogenesis (Figs 1A and B and S1F), have intermediate replication defects. In contrast, *noc3-1-ribo* cells, which are severely defective in ribosome biogenesis (Figs 1A and Figs 1B and S1F), have no obvious replication-initiation defect, with a p1ARS loss rate comparable to WT cells. The phenotypes of these separation-of-function *NOC3* mutants further demonstrate that the replication defects of at least some *NOC3* mutants did not result from the defects in ribosome biogenesis.

Genetic interactions of the separation-of-function mutants with other replication-initiation genes were then studied using the dosage lethality assay (Kroll et al, 1996; Zhai et al, 2010). 17 genes encoding WT initiation proteins controlled by the *GAL* promoter were individually introduced into WT, *noc3-1-ribo*, *noc3-3-rep*, *noc3-9-rep*, and *noc3-145-both* cells. 10-fold serial dilutions of the cells were spotted on glucose- or galactose-containing plates and grown at 25°C (Fig S1G). Some overexpressed proteins, such as *MCM7*, caused dosage lethality phenotypes in WT cells and in the mutants. However, the overexpression of *ORC2*, *ORC3*, *MCM3*, *MCM5*, or *SLD3* impeded the cell growth of *noc3-3-rep* and *noc3-9-rep*, but had only mild to no effects on the other strains that are not severely defective in DNA replication. Moreover, cell growth of *noc3-3-rep*, *noc3-9-rep*, and *noc3-142-both* mutants with overexpressed *DPB11* or *SLD3* was significantly slower compared with the WT and *noc3-1-ribo* mutant. The different sensitivities to the overexpression of the initiation proteins suggest that *noc3-3-rep*, *noc3-9-rep*, and *noc3-142-both* mutants, but not *noc3-1-ribo* cells, are defective in DNA replication. These data are consistent with the results from the plasmid loss assay (Fig 1C).

To further substantiate the separation-of-function properties of the *NOC3* mutants, multicopy suppression assays were performed with *mcm5-1* and *orc5-1* temperature-sensitive mutant cells transformed with pRS425-NOC3, pRS425-noc3-1-ribo, and pRS425-noc3-3-rep multicopy plasmids or pRS425 vector, separately. 10-fold serial dilutions of W3031A, *noc3-1*, *noc3-3*, *mcm5-1*, and *orc5-1* cells without the plasmids were spotted onto YPD plates to show similar temperature sensitivities of different mutants, whereas cells transformed with the plasmids were spotted onto selective plates lacking leucine and grown at the permissive (25°C) or non-permissive (37°C) temperatures to examine cell growth (Fig S1H). Multicopy *NOC3* (WT) suppressed the temperature-sensitive phenotypes of *mcm5-1* (which was also previously reported, Zhang et al, 2002) and *orc5-1*. Similarly, multicopy *noc3-1-ribo* also suppressed the *mcm5-1* and *orc5-1* temperature-sensitive phenotypes; however, multicopy *noc3-3-rep* did not. These data suggest that the *noc3-1-ribo*, but not *noc3-3-rep*, mutant retains DNA replication functions at 37°C. The differential phenotypes of the *NOC3* ts mutants, and different abilities of the multicopy *NOC3* mutant genes to rescue *mcm5-1* and *orc5-1* mutants, collectively demonstrate that Noc3p′s functions in ribosome biogenesis and DNA replication are separable.

### ORC dimerization, pre-RC formation, and DNA replication are impaired in replication-defective *NOC3* mutant cells

Because Noc3p is required for pre-RC formation, which requires ORC dimerization, and Noc3p interacts with four subunits of the Orc1-6p hexamer (Zhang et al, 2002; Huo et al, 2012; Cheung et al, 2019; Wu et al, 2019), we speculated that ORC dimerization, pre-RC formation, and DNA replication may be impaired in replication-defective *NOC3* mutants. To test this, we examined various replication phenotypes in the mutants.

We first examined the chromatin association profiles of ORC and MCM during the M-to-G_1_ transition in different *NOC3* mutants and W303-1A WT cells using the chromatin-binding assay. Substantiated by several other assays including the yeast two-hybrid, co-immunoprecipitation (co-IP), re-ChIP, sucrose gradient, and anchor-away assays, doubling of the chromatin-bound ORC signal, normalized to histone H3, during the M-to-G_1_ transition indicates ORC dimerization (Amin et al, 2020; this study). Similarly, a twofold reduction during the G_1_-to-S transition indicates de-dimerization. The chromatin-binding assay was preferred when we measured the timing of dimerization and de-dimerization of ORC (Amin et al, 2020, and this study) and of Noc3p (this study) in relationship to pre-RC assembly and disassembly, as the assay is especially suited for quantifying the chromatin levels of multiple target proteins and the internal control using the same set of time-point protein samples on the same or parallel immunoblot(s) to minimize variations of the samples and experimental procedures.

**Figure S2.**
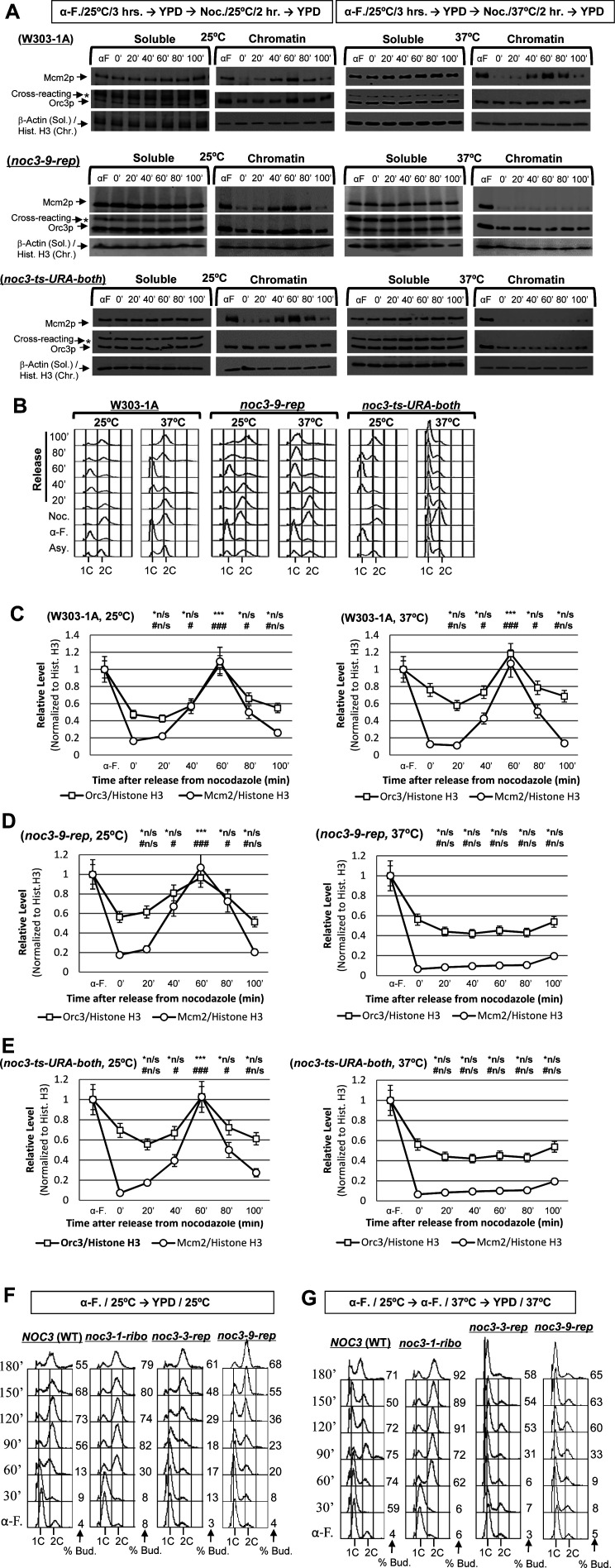
Additional data together with Fig 2 to show that Noc3p is required for ORC dimerization, MCM loading, and cell cycle progression; G_1_/S transition is impeded in *noc3-3-rep* cells at the non-permissive temperature. **(A)** Cells of the indicated strains were pre-synchronized in G1-phase with α-factor (α-F./αF), released into fresh medium, and re-synchronized in nocodazole (Noc./0′) at 25°C and 37°C. Cells were released into fresh medium at 25°C or 37°C and harvested at the indicated time points for chromatin-binding assays to detect the chromatin-bound Mcm2p, Orc3p, and histone H3 (for chromatin)/β-actin (for soluble proteins). *, anti-Orc3 cross-reacting band. **(A, B)** Corresponding flow cytometry analysis for the chromatin-binding assay experiments shown in (A). Asy., asynchronous cells. **(A, C, D, E)** Quantification of the chromatin levels of Orc3p and Mcm2p for the experiments shown in (A) for W303-1A (C), noc3-9-rep (D), and noc3-ts-URA-both (E) cells, presented as the average ± SD of three independent experiments. The signals of Orc3p and Mcm2p were normalized to that of histone H3 at different time points, and the resulting numbers were then further normalized to the G1-phase sample (αF). Statistical analysis was carried out by a paired *t* test, comparing data with those of 0′ time point (Noc.). *, Orc3p; #, Mcm2p; *n/s and #n/s, not significant with *P* > 0.05; */#, *P* < 0.05; **/##, *P* < 0.01; and ***/###, *P* < 0.001. **(F)** α-Factor–synchronized cells of the indicated strains at 25°C were released into fresh medium at 25°C, and cell samples were collected at 30-min intervals. **(G)** α-Factor–synchronized cells of the indicated strains at 25°C were shifted to 37°C for 3 h and then released into fresh medium at 37°C. Cell samples were collected at 30-min intervals.

**Figure 2.**
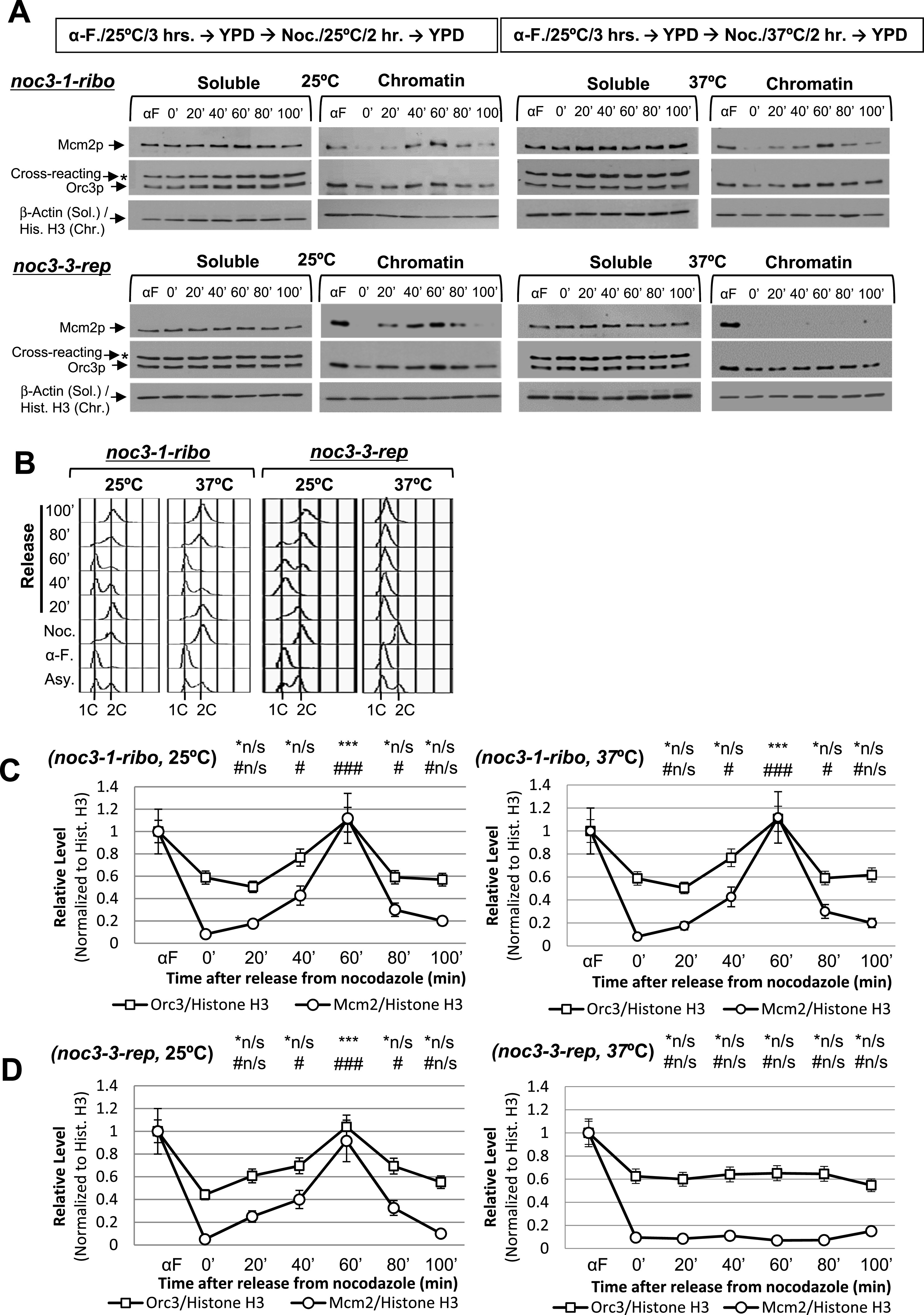
*NOC3* separation-of-function (replication versus ribosome biogenesis) mutants; Noc3p is required for ORC dimerization, MCM loading, and cell cycle progression. **(A)** Indicated mutant cells were pre-synchronized in G_1_-phase with α-factor (α-F.), released into fresh medium, and re-synchronized in nocodazole (Noc./0′) at the permissive (25°C) and non-permissive temperatures (37°C). Cells were released into the cell cycle and harvested at the indicated time points and temperatures for chromatin-binding assay to detect the chromatin-bound levels of Mcm2p, Orc3p, and histone H3 (for the chromatin factions)/β-actin (for the soluble proteins). **(A, B)** Corresponding flow cytometry for the chromatin-binding assay experiments shown in (A). **(A, C, D)** Quantification of the chromatin levels of Orc3p and Mcm2p for the 25°C and 37°C experiments shown in (A) presented as the average ± SD of three independent experiments. The signals of Orc3p and Mcm2p were normalized to that of histone H3 at different time points, and the resulting numbers were then further normalized to the G_1_-phase sample (αF). Statistical analysis was carried out by a paired *t* test (signals of time points versus those in nocodazole). *, Orc3p; #, Mcm2p; not significant (*n/s, #n/s), *P* > 0.05; */#, *P* < 0.05; **/# #, *P* < 0.01; and ***/###, *P* < 0.001.

The cells were pre-synchronized in G_1_-phase with α-factor, released into fresh medium, re-synchronized in M-phase by nocodazole at 25°C or 37°C, again released into the cell cycle, and harvested at different time points and temperatures for chromatin-binding assays. The results show that the chromatin-bound ORC level indeed began to increase before Mcm2p loading and finally doubled in WT (Fig S2A–C) and all *NOC3* mutant cells at 25°C (*noc3-3-rep*, Fig 2A, B, and D; *noc3-9-rep*, Fig S2A, B, and D; *noc3-ts-URA-both*, Fig S2A, B, and E; and *noc3-1-ribo*, Fig 2A–C), and also in WT (Fig S2A–C) and *noc3-1-ribo* (Fig 2A–C) cells at 37°C, indicating normal ORC dimerization and pre-RC formation in these cells under the respective conditions. Cell cycle progression was also normal in all mutant cells at 25°C, with similar flow cytometry profiles to WT cells (Figs 2B and S2B). Contrastingly, the chromatin-bound ORC signal did not increase, Mcm2 did not bind chromatin, and DNA replication did not occur (judged by flow cytometry) in *noc3-3-rep* (Fig 2A, B, and D), *noc3-9-rep* (Fig S2A, B, and D), or *noc3-ts-URA-both* (Fig S2A, B, and E) cells at 37°C, indicating defective ORC dimerization, pre-RC formation, and DNA replication. Most of the cells were budded even at 37°C, indicating that the cyclin-dependent kinases required for cell cycle progression were active. These data indicate that the replication-proficient mutant (*noc3-1-ribo*), but not the replication-deficient mutants (*noc3-3-rep*, *noc3-9-rep*, and *noc3-ts-URA-both*), can support ORC dimerization and pre-RC assembly at the restrictive temperature within a single M-to-G_1_ transition time window.

To corroborate the M-phase block-and-release experiments described above, DNA replication and cell cycle progression of the WT and *noc3-3-rep*, *noc3-9-rep*, and *noc3-1-ribo* mutants after G_1_-phase synchronization and subsequent release were also investigated. Both the WT and *noc3-1-ribo* cells successfully passed the G_1_/S transition and completed S-phase normally at 25°C and 37°C (Fig S2F and G). However, *noc3-3-rep* and *noc3-9-rep* cells showed slow S-phase entry and progression even at 25°C and did not enter S-phase at 37°C, with near-normal budding (Fig S2F and G). The results from both the M-phase and G_1_-phase synchronization-and-release experiments demonstrate that *noc3-3-rep* and *noc3-9-rep* cells are severely defective in DNA replication, different from *noc3-1-ribo* cells, which are only defective in ribosome biogenesis.

**Figure 3.**
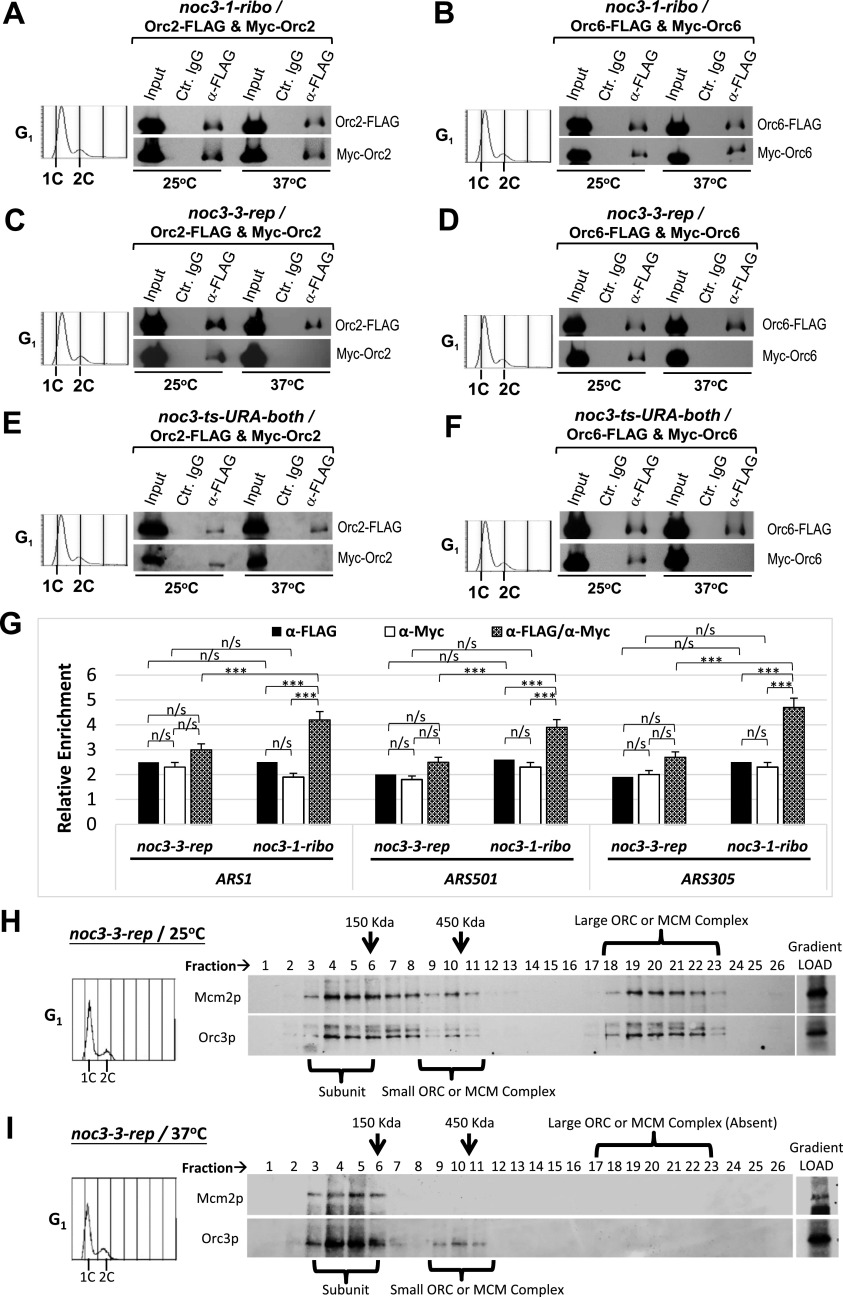
Noc3p is required for ORC dimerization in G_1_-phase, and large molecular forms of endogenous ORC do not exist in G_1_-synchronized *noc3-3-rep* cells at 37°C. **(A, B, C, D, E, F)** Extracts from G_1_-phase–synchronized *noc3-1-ribo* (A, B), *noc3-3-rep* (C, D), and *noc3-ts-URA-both* (E, F) mutant cells expressing Myc-Orc2 and Orc2-FLAG (A, C, E), or Myc-Orc6 and Orc6-FLAG (B, D, F), grown at 25°C or shifted to 37°C, were immunoprecipitated with anti-FLAG antibody or control mouse IgG. Whole-cell extracts (input) and immunoprecipitates by α-FLAG or control IgG were immunoblotted with anti-FLAG and anti-Myc antibodies. **(G)** Sequential ChIP (re-ChIP) assays were performed with G_1_-synchronized *noc3-1-ribo* or *noc3-3-rep* cells co-expressing Orc6-FLAG and Myc-Orc6 shifted to 37°C. Extracts were first immunoprecipitated with anti-FLAG antibody. The anti-FLAG ChIP chromatin immunoprecipitates were eluted and then immunoprecipitated with anti-Myc antibody in the second ChIP. Real-time PCR was performed using primers to quantify *ARS1*, *ARS1* + 2.5 kb, *ARS501*, *ARS501* + 11 kb, *ARS305*, and *ARS305* + 8 kb. The relative enrichment (ARS normalized to non-ARSs) was calculated and averaged from three independent experiments. Data are presented as the mean ± SD. Statistical analysis was carried out by one-Way ANOVA and Dunnett’s multiple comparison test. Not significant (n/s), *P* > 0.05; *, *P* < 0.05; **, *P* < 0.01; and ***, *P* < 0.001. **(H, I)** *noc3-3-rep* mutant cells synchronized in G_1_-phase at 25°C (H) and those shifted to 37°C (I) were cross-linked, harvested, and used to prepare total protein extracts for 20–60% sucrose gradient analysis. Alkaline phosphatase (150 kD) and β-galactosidase (450 kD) were applied as protein markers. The resulting 26 fractions and the gradient load from each cell sample were resolved by 10% SDS–PAGE and were immunoblotted with anti-Orc3 and anti-Mcm2 antibodies. Flow cytometry was used to determine the cell cycle distribution of the cells. The immunoblots are representative images from one of the three independent experiments that produced similar results.

The role of Noc3p in ORC dimerization was further confirmed using co-IP assays with G_1_-phase–synchronized *NOC3* ts mutant cells (Fig 3A–F). Positive co-IP between Orc6-FLAG and Myc-Orc6, and between Orc2-FLAG and Myc-Orc2, by anti-FLAG antibody, but not the control IgG, was detectable in the replication-proficient *noc3-1-ribo* cells (Fig 3A and B). In contrast, *noc3-3-rep* (Fig 3C and D) and *noc3-ts-URA* (Fig 3E and F) cells gave negative co-IP results at 37°C, confirming that ORC dimerization was impaired in the two DNA replication-deficient *NOC3* mutant strains.

Moreover, Orc6-FLAG and Myc-Orc6 could be detected at the same origins of replication in the replication-proficient *noc3-1-ribo* cells, but not in the replication-deficient *noc3-3-rep* cells, by regular chromatin immunoprecipitation (ChIP, which detects origin binding by Noc3p in all forms) and sequential ChIP (or re-ChIP, which detects co-occupancy of an origin by two Noc3p molecules; Geisberg et al, 2004; Huo et al, 2012; Amin et al, 2020) assays with α-factor–synchronized G_1_-phase cells at 37°C (Fig 3G). These data support the conclusion that ORC dimerization at ARSs requires Noc3p. Taken together, the data from plasmid loss, co-IP, chromatin-binding, and re-ChIP assays and flow cytometry demonstrate that Noc3p is required for ORC dimerization, pre-RC formation, and DNA replication, independent of Noc3p’s role in ribosome biogenesis.

**Figure S3.**
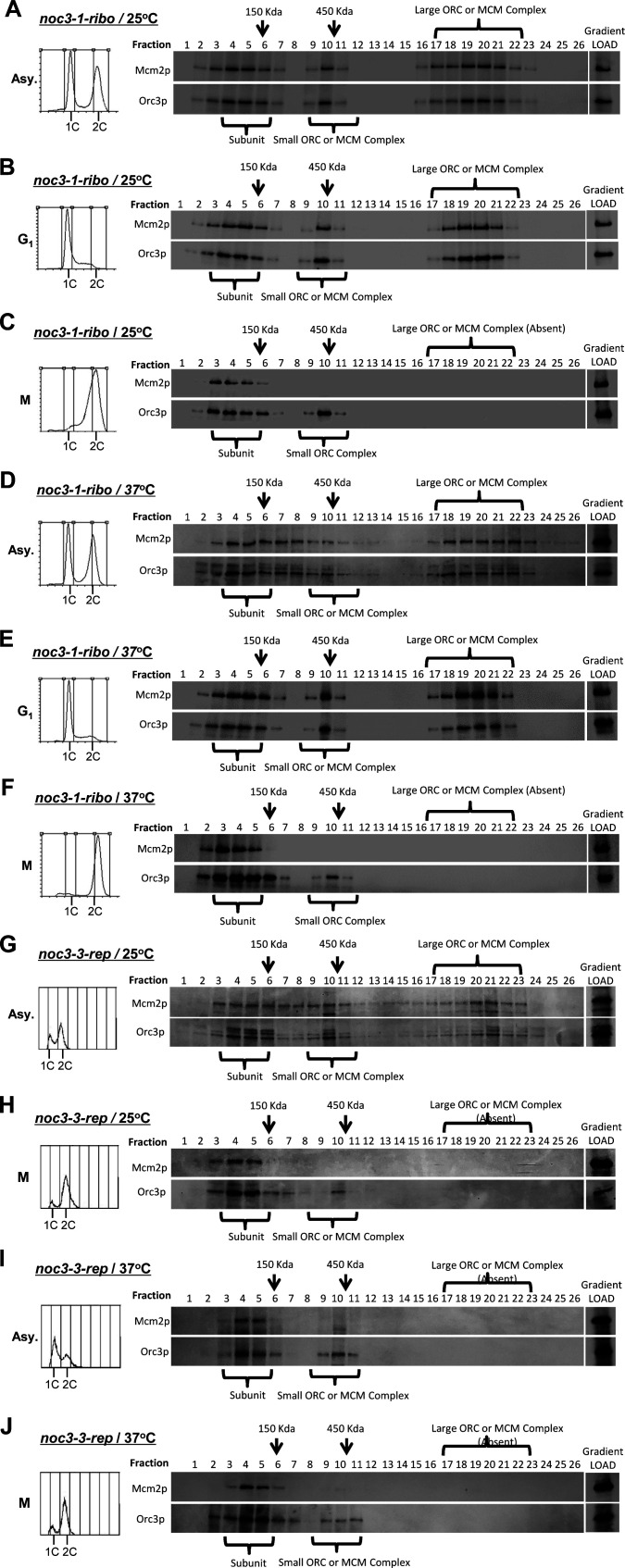
Additional data together with Fig 3 to show that Noc3p is required for the formation of larger molecular forms of endogenous ORC in asynchronous and G_1_-phase cells but not M-phase cells **(A, B, C, D, E, F, G, H, I, J)** Mutant noc3-1-ribo cells growing asynchronously or synchronized (G1-phase/M-phase) at 25°C (A, B, C) and those shifted to 37°C (D, E, F), and noc3-3-rep mutant cells grown asynchronously or synchronized in M-phase at 25°C (G, H) and those shifted to 37°C (I, J) were cross-linked with formaldehyde, harvested, and used to prepare total protein extracts for 20–60% sucrose gradient analysis. Alkaline phosphatase (150 kD) and β-galactosidase (450 kD) were applied as protein markers. The resulting 26 fractions and the gradient load from each cell sample were run on 10% SDS–PAGE and were immunoblotted with anti-Orc3 and anti-Mcm2 antibodies. Flow cytometry was used to determine the cell cycle distribution of the cells. The immunoblots are representative images from three independent experiments that produced similar results.

To further substantiate the role of Noc3p in ORC dimerization, we performed sucrose gradient analysis to examine the molecular forms of the endogenous ORC in *noc3-1-ribo* and *noc3-3-rep* cells, with a small (∼450 kD) and a large (>1,000 kD) complex indicating the ORC monomers and dimers, respectively (Amin et al, 2020). The results show that ORC, represented by Orc3p, from asynchronous (Asy.; Fig S3A and G) and G_1_-phase (Figs 3H and S3B) *noc3-1-ribo* and *noc3-3-rep* cells grown in the presence of α-factor at 25°C sedimented as subunits (fractions 3–6: <150 kD), a small ORC (fractions 8–12: ∼450 kD), and a large ORC (fractions 17–22), consistent with ORC dimerization. The ORC from M-phase *noc3-1-ribo* and *noc3-3-rep* cells grown in the presence of nocodazole at 25°C sedimented as subunits (fractions 3–6: <150 kD) and a small ORC (fractions 8–12: ∼450 kD), whereas the larger ORC was absent (Fig S3C and H), indicating the absence of ORC dimers in M-phase cells synchronized by nocodazole. As expected, MCM, represented by Mcm2p, from asynchronous (Fig S3A and G) and G_1_ (Figs 3H and S3B) *noc3-1-ribo* and *noc3-3-rep* cells grown at 25°C sedimented as subunits (fractions 3–6: <150 kD), a small MCM complex (fractions 8–11: ∼450 kD), and a large MCM complex (fractions 16–21), consistent with pre-RC formation. The MCM from M-phase *noc3-1-ribo* and *noc3-3-rep* cells grown at 25°C sedimented only as subunits (fractions 3–5: <150 kD), whereas both the small and larger MCM complexes were absent (Fig S3C and H), as expected for the post-RC state. These results are as expected for ORC-forming double-hexamers in G_1_-phase and single-hexamers in M-phase in both *noc3-1-ribo* and *noc3-3-rep* cells at 25°C.

At 37°C, ORC and MCM proteins in asynchronous (Fig S3D), G_1_-phase (Fig S3E), and M-phase (Fig S3F) *noc3-1-ribo* cells behaved in the same way as *noc3-1-ribo* and *noc3-3-rep* cells at 25°C. These results are consistent with ORC-forming double-hexamers in G_1_-phase and single-hexamers in M-phase even at 37°C. Conversely, in *noc3-3-rep* cells at 37°C, the larger ORC was absent, whereas the small ORC persisted in asynchronous (Fig S3I), G_1_-phase (Fig 3I), and M-phase (Fig S3J), indicating ORC dimerization failure. MCM sedimented only as subunits (fractions 3–5: <150 kD), whereas both the small and large MCM complexes were absent, indicating pre-RC formation failure. Together, the results from the studies of the *NOC3* mutants demonstrate the separation of function of the *NOC3* mutants and confirm that ORC dimerization, pre-RC formation, and DNA replication require Noc3p in vivo.

### Noc3p self-interacts and dimerizes in a cell cycle–regulated manner, and two Noc3p molecules co-occupy the same ARSs

Previously, we performed comprehensive yeast two-hybrid analyses to examine the pair-wise interactions among budding yeast and human pre-RC proteins (Huo et al, 2012; Wu et al, 2019). In addition to many inter-subunit interactions identified, self-interactions, that is, the interactions between molecules of the same subunit, were particularly noteworthy. Importantly, self-interactions of MCM subunits match the reported dimerization of MCM (Lei et al, 1996; Evrin et al, 2009; Remus et al, 2009), and self-interactions of ORC lead to the discovery of ORC dimerization (Amin et al, 2020).

**Figure 4.**
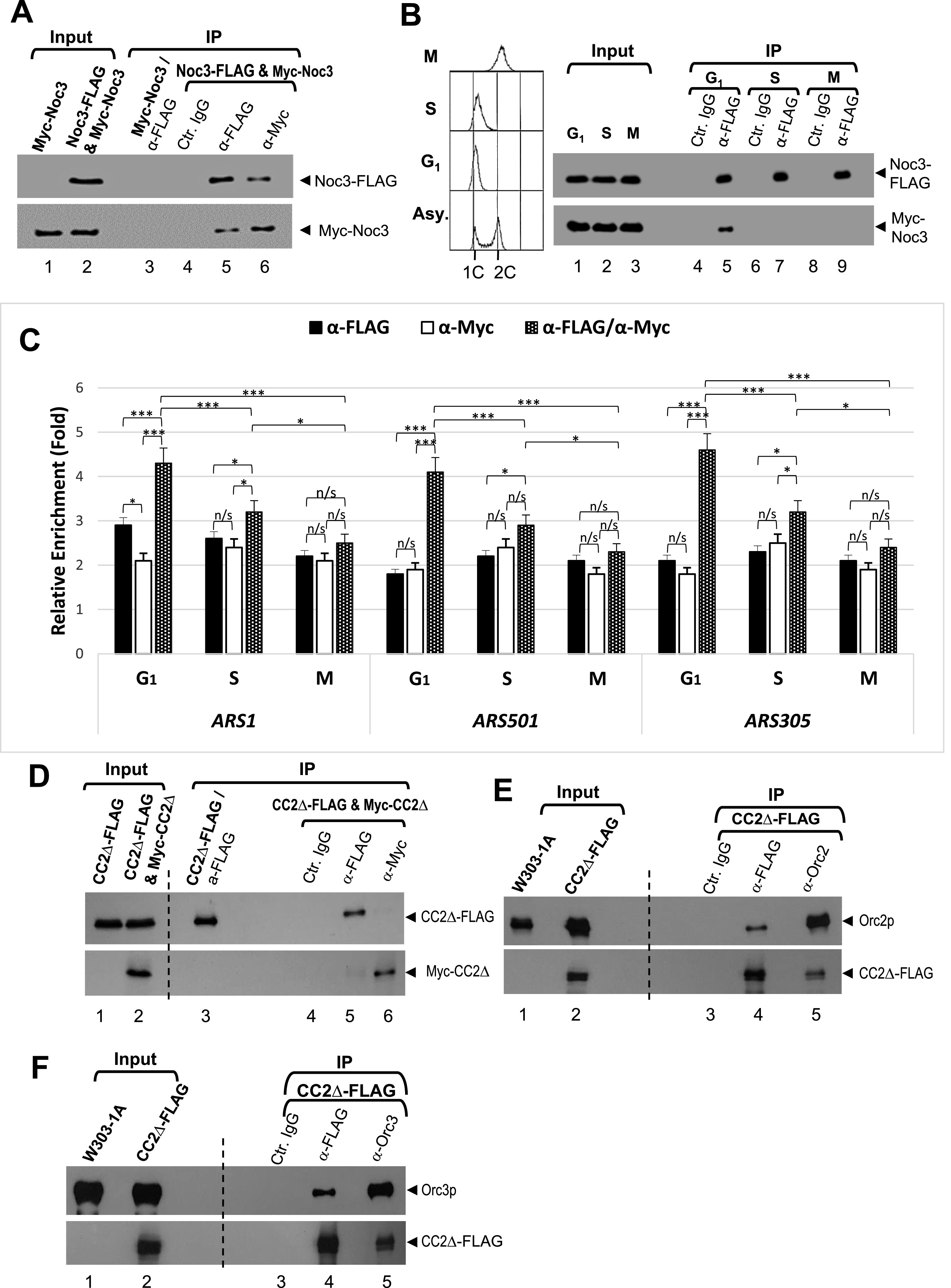
Noc3p self-interacts and dimerizes in a cell cycle–regulated manner, and deletion of the Noc3p self-interaction domain impairs self-interaction but not interaction with ORC. **(A)** Extracts from cycling cells expressing Myc-Noc3 and Noc3-FLAG were immunoprecipitated with anti-FLAG antibody, anti-Myc antibody, or control mouse IgG. Whole-cell extracts (input) and immunoprecipitates (IP) were immunoblotted with anti-FLAG and anti-Myc antibodies. Note that the Myc-Noc3p signal in lane 1 is slightly distorted. **(B)** Extracts from cells expressing Noc3-FLAG and Noc3-Myc in G_1_-, S-, or M-phase were immunoprecipitated by anti-FLAG and probed for Noc3-FLAG and Noc3-Myc. Flow cytometry data for the co-IP are also shown. **(C)** Sequential ChIP (re-ChIP) assays were performed with G_1_-, S-, or M-phase–synchronized cells co-expressing Noc3-FLAG and Myc-Noc3. Extracts were first immunoprecipitated with anti-FLAG antibody. The anti-FLAG chromatin immunoprecipitates were then eluted and immunoprecipitated with anti-Myc antibody in the second ChIP. Real-time PCR was performed using primers to quantify *ARS1*, *ARS1* + 2.5 kb, *ARS501*, *ARS501* + 11 kb, *ARS305*, and *ARS305* + 8 kb. The relative enrichment (ARS normalized to non-ARSs) was calculated and averaged from three independent experiments. Data are presented as the mean ± SD. Statistical analysis was carried out by one-Way ANOVA and Dunnett’s multiple comparison test. Not significant (n/s), *P* > 0.05; *, *P* < 0.05; **, *P* < 0.01; and ***, *P* < 0.001. **(D, E, F)** Extracts from asynchronous cells expressing Noc3-CC2∆-FLAG and Myc-Noc3-CC2∆ (D), and those expressing Noc3-CC2∆-FLAG only (E, F), were immunoprecipitated with the indicated antibodies or control mouse IgG. Whole-cell extracts (input) and immunoprecipitates (Co-IP) were immunoblotted with the indicated antibodies.

Another self-interaction was that of budding yeast Noc3p (Huo et al, 2012; Wu et al, 2019). Interestingly, human NOC3 was also found to have self-interactions (Cheung et al, 2019). To verify these findings, reciprocal co-IP assay was performed in budding yeast cells expressing two differently tagged versions of Noc3p (Noc3-FLAG and Myc-Noc3). DNase I–digested yeast cell extracts were immunoprecipitated separately with anti-Myc and anti-FLAG antibodies. The immunoprecipitates were immunoblotted using anti-Myc and anti-FLAG antibodies. The results show that Noc3-FLAG and Myc-Noc3 could be co-immunoprecipitated by either anti-Myc or anti-FLAG antibodies, whereas the negative controls gave negative results (Fig 4A), confirming Noc3p-Noc3p self-interactions in vivo. This self-interaction has also recently been suggested by affinity capture mass spectrometry in an in vitro cryo-EM study that examined ribosome biogenesis complexes (Zhou et al, 2019).

To determine whether Noc3p dimerization is cell cycle–dependent, similar to ORC dimerization (Amin et al, 2020), co-IP assays were performed to examine Noc3p self-interactions in yeast cells synchronized in G_1_-, S-, or M-phase by α-factor, hydroxyurea, or nocodazole, respectively. The results indicate that Noc3p self-interacts in G_1_-phase, but not in S- or M-phase (Fig 4B). These findings suggest that Noc3p-Noc3p self-interactions are coupled to the cell cycle. The G_1_-phase–specific Noc3p-Noc3p co-IP also demonstrates that the co-IP results in Fig 4A were unlikely due to indirect, spurious protein association or artifacts.

To substantiate co-occupancy of two Noc3p subunits on the same replication origins, regular (or single-) ChIP and re-ChIP assays were performed with G_1_-, S-, or M-phase–synchronized cells expressing Noc3-FLAG and Myc-Noc3p. The results indicate that the single-ChIP assay enriched ARSs relative to non-ARS control regions, whereas the re-ChIP assay further enriched the ARSs compared with the single-ChIP assays (Fig 4C). These data suggest that like ORC, two Noc3p molecules co-occupy the same ARSs in G_1_-phase. Noc3p co-occupancy at the same ARSs was also observable in S-phase (attributable to late-firing origins), but at a much lower level than G_1_-phase, whereas co-occupancy was not observed in M-phase.

### The Noc3p self-interaction domain is essential for Noc3p dimerization and cell viability

**Figure S4.**
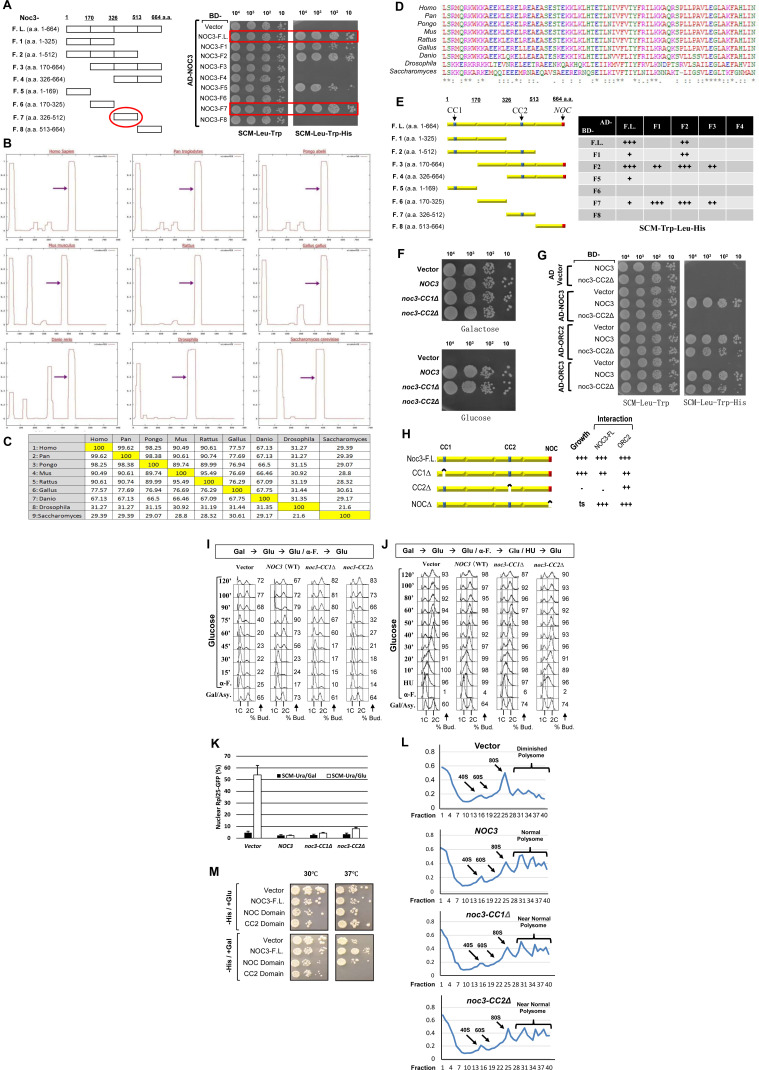
Additional data together with Fig 4 to show that the CC2 domain of Noc3p is conserved across species; CC2 is required for Noc3p self-interaction but not for interaction with ORC; CC2 is essential for S-phase entry but not DNA replication elongation; and ribosome biogenesis is not affected by the Noc3p truncation or the *NOC3* endogenous gene shutoff within the experimental time window. **(A)** Schematic diagram of the NOC3 fragments studied and mapping of the Noc3p self-interaction domains by the yeast two-hybrid assay. Interactions between pairs of BD- and AD-fusion proteins were examined by yeast two-hybrid analysis on SCM-Trp-Leu (non-selective for the protein–protein interactions examined) and SCM-Trp-Leu-His (selective for the protein–protein interactions examined) plates. As negative controls, the host yeast cells were co-transformed with the empty BD or AD vector together with the individual fusion proteins as indicated. **(B)** Results of MultiCoil analysis of NOC3 from different species. Arrowheads indicate the conserved C-terminal coiled-coil domains (CC2). **(C)** Similarity comparison of NOC3 across different species. **(D)** Multiple alignments of the C-terminal coiled-coil region of NOC3 from different species: *H. sapiens* (Homo), *P. troglodytes* (Pan), *P. pygmaeus* (Pongo), *M. musculus* (Mus), *R. norvegicus* (Rattus), *G. gallus* (Gallus), *D. rerio* (Danio), *D. melanogaster* (Drosophila), and *S. cerevisiae* (Saccharomyces). **(E)** Schematic diagram of the Noc3p fragments studied. A summary table lists the relative strengths of the respective interactions observed by the host yeast cell growth on SCM-Trp-Leu-His plates. **(F)** 10-fold serial dilutions of GAL-NOC3 cells harboring the empty vector or the plasmid expressing WT Noc3p, noc3-CC1Δ, or noc3-CC2Δ. The cells were spotted onto galactose- or glucose-containing plates. **(G)** Yeast two-hybrid assays for the interactions of BD-NOC3 and BD-Noc3-CC2Δ with AD-Noc3p, AD-Orc2p, and AD-Orc3p. **(H)** Schematic diagram of the NOC3 domain deletion mutants studied, the resulting growth phenotypes of the cells expressing the WT or Noc3p deletion mutants, and the relative strengths of the respective interactions of the WT or mutant proteins observed by the host yeast cell growth on SCM-Trp-Leu-His plates. **(I)** Expression of the endogenous Noc3p in GAL-NOC3 cells, containing the empty vector, WT NOC3, noc3-CC1Δ, or noc3-CC2Δ plasmids, was shut down in glucose-containing medium for 4 h before the cells were synchronized in G1-phase by α-factor (α-F.). Cells were then released into fresh glucose-containing medium. Flow cytometry was performed with the cell samples taken at the indicated time points. Asy., asynchronous cells. **(J)** GAL-NOC3 cells containing the empty vector or the plasmid expressing Noc3p, Noc3-CC1Δ, or Noc3-CC2Δ were grown in glucose-containing medium for 1.5 h (to suppress the Noc3p expression from GAL-NOC3) before being synchronized in G1-phase with α-factor. The cells were then released from the G1-phase block into early S-phase in hydroxyurea (HU)-containing medium with glucose for 2.5 h. Afterward, the cells were released into fresh medium containing glucose. Flow cytometry was performed with the cell samples taken at the indicated time points. **(K)** Quantification of the nuclear-accumulated Rpl25-GFP in the indicated strains. **(I)** GAL-NOC3 cells containing the indicated plasmids were cultured as described in (I), and the Rpl25-GFP localization was examined before and after the cells were shifted to glucose-containing medium. **(F, G, K, L)** Ribosome profiles (OD260) for the strains described in (F, G, K) after the cells were shifted to glucose-containing medium. Equal amounts of cell lysates were fractionated through on 20–60% sucrose gradients. The 40S small subunit, 60S large subunit, and 80S mono-ribosome and poly-ribosome were indicated by arrows. **(M)** NOC domain deletion ts mutant cells containing the empty vector or the plasmid expressing the WT Noc3p, Noc3-NOCΔ mutant, or noc3-CC2Δ mutant proteins were grown on galactose- or glucose-containing plates at the indicated temperatures.

To further understand the biological significance of Noc3p self-interaction, *NOC3* was dissected into fragments to identify the self-interacting region by yeast two-hybrid analysis. The results show that at least two regions of Noc3p, Noc3-F5 and Noc3-F7, are involved in Noc3p self-interaction, with Noc3-F7 probably contributing more than Noc3-F5 (Fig S4A).

The two domains were analyzed using computation COILS and MultiCoil algorithm predictions (Lupas et al, 1991) (Fig S4B). One coiled-coil motif was identified in Noc3-F5 (aa 39–49, referred to as CC1), and another, in Noc3-F7 (aa 325–512, referred to as CC2) (Fig S4B). Although Noc3p is not a highly conserved protein across different species (Fig S4C), surprisingly, all seven Noc3p homologs analyzed were predicted to contain a coiled-coil motif at almost the same position as CC2 with high similarities (Fig S4B and D), suggesting that the CC2 may perform some important functions.

We also examined the pair-wise inter-domain interactions of the Noc3p fragments. The CC2-containing fragment F7 had interactions with the full-length protein, and F3 and F2 fragments, all of which contained CC2 (Fig S4E). Interestingly, F7 also interacted with the F1 fragment, which does not contain CC2 but contains a region that is adjacent to CC2, which is also present within the F3 and F2 fragments that have interactions with CC2, suggesting that both regions are important for Noc3p self-interaction. The CC1-containing fragment F5 only interacted with the full-length protein, but not the N- or C-terminal fragments. The *NOC* domain–containing fragment F8 had no interactions with the other fragments. These results suggest that CC2 is the major part of the Noc3p self-interaction domain.

To investigate the functions of the coiled-coil motifs on Noc3p, two mutants were constructed by deleting the CC1 and CC2 domains (named *noc3-CC1Δ* and *noc3-CC2Δ*, respectively). Cells with *noc3-CC1Δ* were able to grow when the expression of the *GAL-NOC3* was suppressed by glucose, similar to cells expressing WT *NOC3* (Fig S4F), indicating that *noc3-CC1Δ* is functional. On the contrary, *noc3-CC2Δ* did not support cell viability, similar to the vector control (Fig S4F). These results suggest that CC1 is not essential, whereas CC2 is required for cell proliferation, consistent with the conservation of CC2 of Noc3p.

Because Noc3-F7, which contains CC2, interacts with full-length Noc3p and the deletion of CC2 impairs cell survival, we examined whether the *noc3-CC2Δ* mutant failed to interact with Noc3p by yeast two-hybrid assays. As expected, Noc3-CC2Δ completely lost its interaction with WT Noc3p, but still interacted with Orc2p and Orc3p (Fig S4G), suggesting that the conformation of Noc3-CC2Δ is largely maintained. On the contrary, Noc3-CC1Δ interacted with Noc3p (Fig S4H), consistent with its ability to support cell growth. These results, together with those from the molecular dissection of Noc3p, indicate that the CC2 domain of Noc3p is conserved and essential for Noc3p self-interaction.

The results from the yeast two-hybrid analyses were further corroborated by the lack of interactions in reciprocal co-IP between Noc3-CC2Δ-FLAG and Myc-Noc3-CC2Δ by anti-Myc or anti-FLAG antibodies from yeast cell extracts (Fig 4D). These results confirmed that the Noc3-CC2Δ mutant was unable to support Noc3p self-interactions (Fig 4A). Noc3-CC2Δ-FLAG was still able to interact with Orc2p (Fig 4E) and Orc3p (Fig 4F). Together, these data strongly suggest that CC2 is the major part of the self-interaction domain of Noc3p.

The effects of CC1 and CC2 deletion on cell cycle progression were then examined by flow cytometry. *GAL-NOC3* cells expressing WT *NOC3*, *noc3-CC1Δ*, or the *noc3-CC2Δ* were released from G_1_-phase into fresh glucose–containing medium to suppress *GAL-NOC3* expression. The results showed that cells expressing WT *NOC3* started DNA replication at 30 min after release, whereas cells expressing *noc3-CC1Δ* entered S-phase with a slight delay of 15 min (Fig S4I). However, S-phase was significantly impaired in cells expressing *noc3-CC2Δ*, which is similar to the cells with the empty vector (Fig S4I). The eventual S-phase entry (at 90 min) by the *noc3-CC2Δ* or the empty vector control cells was attributable to *GAL-NOC3* leaky expression.

We ruled out the possibility that the DNA replication defects in the *noc3-CC2Δ* mutant were caused by replication elongation defects, as cells expressing Noc3-CC2*Δ* could progress through S-phase normally after release from an S-phase block by hydroxyurea (Fig S4J). This is consistent with the previous report that Noc3p is not required for S-phase progression (Zhang et al, 2002). We also ruled out the possibility that the DNA replication defect in the *noc3-CC2Δ* mutant was caused by ribosome biogenesis failure, as ribosome biogenesis in all of the cells, except those with the empty vector, was more or less normal, as indicated by the predominately cytoplasmic Rpl25-GFP localization (Fig S4K) and the normal poly-ribosome profiles (Fig S4L). This also provides another example of separation of function by the *noc3-CC2Δ* mutant.

Interestingly, deletion of the C-terminal *NOC* domain (Milkereit et al, 2001), although not disrupting Noc3p′s interactions with Noc3p and Orc2p (Fig S4H), generated a ts mutant (Fig S4M), suggesting that the *NOC* domain is essential for the Noc3p function at 37°C. We used this phenotype to further demonstrate the function of the Noc3p CC2 domain. The cell growth defects at 37°C were rescued by WT *NOC3*, but not *noc3-CC2Δ* mutant, further supporting that the CC2 domain is essential for the Noc3p function. Taken together, our data from the domain analyses demonstrate that the Noc3p CC2 motif is essential for Noc3p dimerization and DNA replication in a ribosome biogenesis–independent manner, further substantiating that the functions of Noc3p in DNA replication and ribosome biogenesis are separable.

### Free Noc3p binds to chromatin-bound Noc3p to form Noc3p dimers in late M-phase before MCM loading, and Noc3p de-dimerizes in S-phase

**Figure 5.**
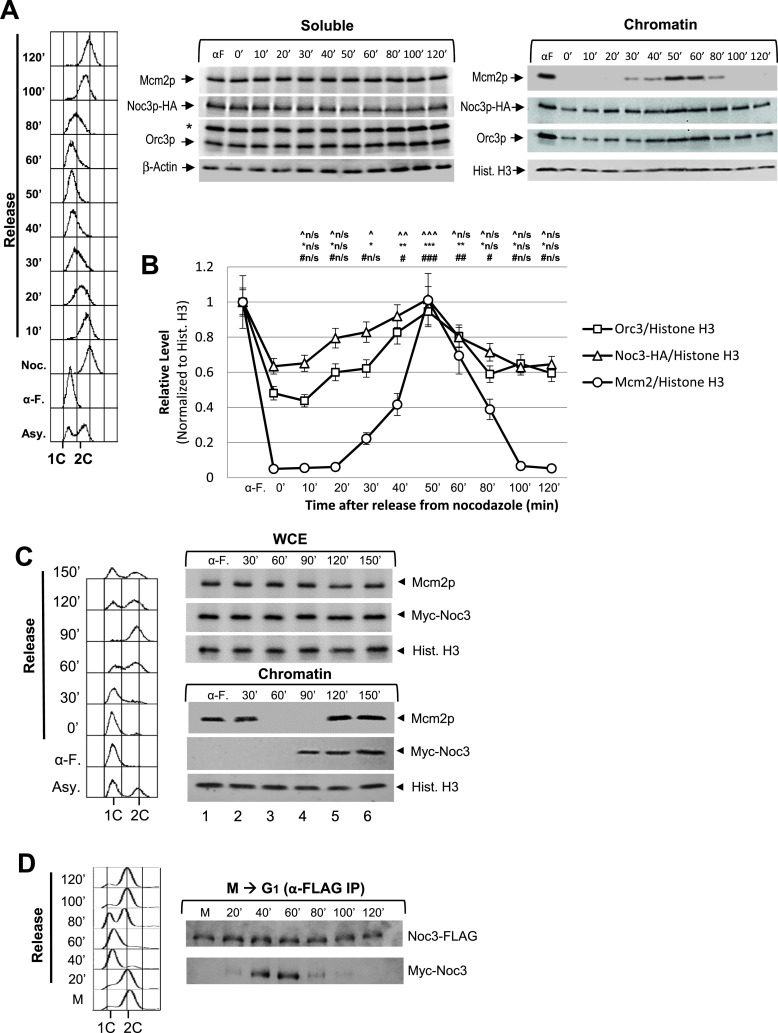
Free Noc3p loads onto chromatin to form double-hexamers in late M-phase before MCM loading. **(A)** *NOC3-HA* cells were pre-synchronized in G_1_-phase with α-factor (α-F./αF) and then released into fresh medium. The cells were then re-synchronized in M-phase with nocodazole (Noc./0′). The cells were subsequently released into the cell cycle for chromatin-binding assay to detect the chromatin-bound Mcm2p, Noc3p-HA, Orc3p, and histone H3 (for chromatin) or β-actin (for soluble proteins). *, anti-Orc3 cross-reacting band. **(A, B)** Quantification of the chromatin levels of Mcm2p, Noc3p-HA, and Orc3p for the experiments shown in (A), presented as the average ± SD of three independent experiments. The signals of Orc3p, Noc3p-HA, and Mcm2p were normalized to that of histone H3 at different time points, and the resulting numbers were then further normalized to the G_1_-phase sample (αF). Statistical analysis was carried out by a paired *t* test, comparing data with those of the 0′ time point (Noc.). (^, Noc3p-HA; *, Orc3p; #, Mcm2p; not significant [^n/s, *n/s, #n/s], *P* > 0.05; ^/*/#, *P* < 0.05; ^^/**/##, *P* < 0.01; and ^^^/***/###, *P* < 0.001). **(C)** *GAL-Myc-NOC3* cells were synchronized in G_1_-phase, induced to express Myc-Noc3, and then released into the cell cycle. Samples were collected at the indicated time points. Whole-cell extracts and DNase I–solubilized chromatin fractions were immunoblotted for Mcm2p, Myc-Noc3, and histone H3. **(D)** W303-1A (WT) cells expressing Noc3-FLAG and Myc-Noc3 were synchronized in M-phase and released into G_1_-phase in fresh medium containing α-factor. Cell samples were harvested at the indicated time points for co-IP. The cell cycle stages as marked were determined by flow cytometry.

To determine the time in the cell cycle when free Noc3p binds chromatin to form dimers, the chromatin association patterns of Noc3p, ORC, and MCM were studied across the cell cycle. Nocodazole-blocked, M-phase–synchronized cells were released into fresh medium and harvested at different time points for analysis. The chromatin-bound Noc3p and ORC levels began to increase 10–20 min after release (Fig 5A and B). Mcm2p loading started at 30 min after release. As a double of the ORC level on chromatin indicates ORC dimerization (Amin et al, 2020; and as discussed in Fig 2), the data shown in Fig 5A and B suggest that Noc3p dimerization and ORC dimerization occur before MCM loading.

**Figure S5.**
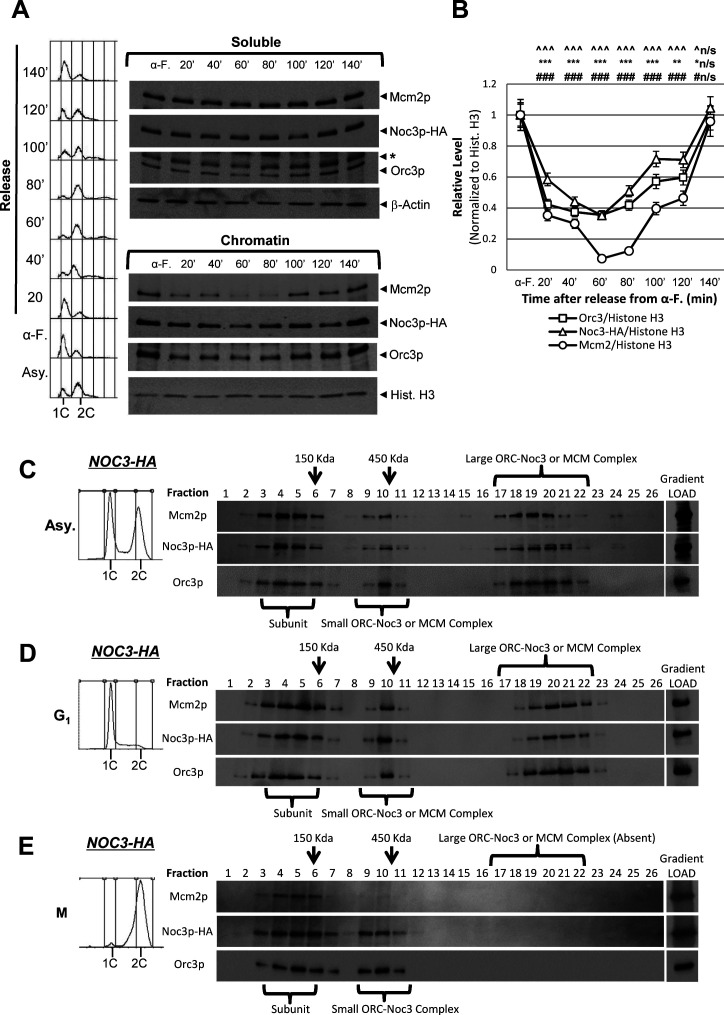
Additional data together with Fig 5 to show that Noc3p self-interacts and dimerizes in a cell cycle–regulated manner, and the existence of larger molecular forms of endogenous Noc3p in asynchronous and G_1_-phase cells, but not M-phase cells. **(A)** NOC3-HA cells were arrested in G1-phase with α-factor and then released into fresh medium. Cells from equal culture volumes were harvested at the indicated time points for chromatin-binding assay to detect the chromatin-bound Mcm2p, Noc3p-HA, Orc3p, and histone H3 (for chromatin) or β-actin (for supernatant). *, anti-Orc3 cross-reacting band. **(A, B)** Quantification of the chromatin levels of Orc3p, Mcm2p, and Noc3-HA for the experiments shown in (A), presented as the average ± SD of three independent experiments. The signals of Orc3p, Noc3p-HA, and Mcm2p were normalized to that of histone H3 at different time points, and the resulting numbers were then further normalized to the G1-phase sample (αF). Statistical analysis was carried out by a paired *t* test (each time point versus that in α-factor block). ^, Noc3-HA; *, Orc3p; #, Mcm2p; not significant (^n/s, *n/s, #n/s), *P* > 0.05; ^, *, #, *P* < 0.05; ^^, **, # #, *P* < 0.01; and ^^^, ***, ###, *P* < 0.001. **(C, D, E)** NOC3-HA cells growing asynchronously or synchronized (G1-phase or M-phase) were cross-linked, harvested, and used to prepare total protein extracts for 20–60% sucrose gradient analysis. Alkaline phosphatase (150 kD) and β-galactosidase (450 kD) were applied as protein markers. The resulting 26 fractions and the gradient load from each cell sample were run on 10% SDS–PAGE and were immunoblotted with anti-HA, anti-Orc3, and anti-Mcm2 antibodies. Flow cytometry was used to determine the cell cycle distribution of the cells. The immunoblots are representative images from three independent experiments that produced similar results.

The de-dimerization of Noc3p, ORC, and MCM was studied using cells released from a G_1_-phase block. α-factor–synchronized cells were released into fresh medium and harvested at different time points for analysis. S-phase progression (from 40 to 80 min after release) was coupled to a steady decrease in chromatin-bound Orc3p and Noc3p, normalized to histone H3, eventually decreasing by twofold in late S-phase (Fig S5A and B), consistent with ORC de-dimerization (Amin et al, 2020) and Noc3p de-dimerization. Mcm2p chromatin disassociation occurred during S-phase, as expected. In addition, the amount of chromatin-associated Orc3p and Noc3p started to increase at 60–80 min, reaching a twofold level in late M-phase, preceding Mcm2p loading (100′–140′; Fig S5A and B). Together with the data from the M-phase release experiment (Fig 5A and B), our data indicate that dimerization of Noc3p and of ORC occurs around the same time, ahead of MCM loading, at the M-to-G_1_ transition, and that de-dimerization of Noc3p and of ORC happens in S-phase.

To substantiate these findings, we examined the cell cycle chromatin-association pattern of Myc-Noc3, which was induced to express from the *GAL* promoter in G_1_-phase. Although the ectopically expressed Noc3p was present in whole-cell extracts across the cell cycle, it only associated with the chromatin in late M-phase (90 min, before MCM loading at 120 min) rather than in G_1_-, S-, G_2_-, or early- to mid-M-phase (Fig 5C). The chromatin-binding patterns of the endogenous Noc3p and ectopically expressed Myc-Noc3 (Fig 5A–C) indicate that Noc3p, like ORC, is continuously bound to the chromatin throughout the cell cycle and that free Noc3 protein associates with the chromatin-bound Noc3p to form a dimer only in the late M-phase before MCM loading.

To further corroborate chromatin-binding assay results, we performed co-IP and found that Noc3-FLAG and Myc-Noc3 interacted at 20–60 min after cells were released from the M-phase block (Fig 5D). These results confirm the timing of Noc3p dimerization revealed by the chromatin-binding assays. Negative co-IP results were observed at 60–80 min when the cells were in S-phase, indicating Noc3p de-dimerization during S-phase.

To further substantiate the association of Noc3p with ORC, in both monomer and dimer forms, we performed sucrose gradient analysis with *NOC3-HA* cells grown at 25°C. The results show that Noc3-HA co-sedimented with ORC (represented by Orc3p) in the gradients when the extracts were from asynchronous (Fig S5C), G_1_-phase (Fig S5D), and M-phase (Fig S5E) cells, whereas different forms of MCM, represented by Mcm2p, behaved as expected for the respective pre-RC state in G_1_-phase and post-RC state in M-phase. Although the sucrose gradient experiment does not explicitly demonstrate whether Noc3-HA in the large complex with ORC has two Noc3-HA molecules or not, our data from other experiments of this study demonstrate that Noc3p dimerizes in a cell cycle–dependent manner. Therefore, the corresponding cell cycle–specific patterns of Noc3-HA and ORC in the sucrose gradients suggest that the small ORCs contain Noc3-HA monomers, whereas the large ORCs contain Noc3-HA dimers.

Taken together, the data in Figs 5 and S5 are consistent with a semi-conservative model of the Noc3p dimerization cycle, similar to and coupled with that of ORC; that is, after DNA replication, the origin-bound Noc3p dimer, together with an ORC dimer, separates into two Noc3p-ORCs, each binding one of the two nascent sister origins until late M-phase, during which free Noc3p and ORC proteins associate with the chromatin-bound Noc3p-ORC to re-form the (Noc3p-ORC)_2_ dimer before MCM loading.

### Noc3p dimerization is required for ORC dimerization, pre-RC formation, DNA replication, and cell proliferation

Given that chromatin-unbound Noc3p associated with chromatin-bound Noc3p for Noc3p dimerization in late M-phase, depletion of free Noc3p before pre-RC formation should abrogate Noc3p dimerization. The anchor-away system (Haruki et al, 2008) was used to deplete free Noc3p from the nucleus in cells containing FRB-tagged Noc3p. We have previously used this method to study ORC dimerization by depleting FRB-tagged ORC proteins from the nucleus through their interactions with a ribosomal protein tagged with an FRB-interacting “anchor” in the presence of rapamycin.

**Figure S6.**
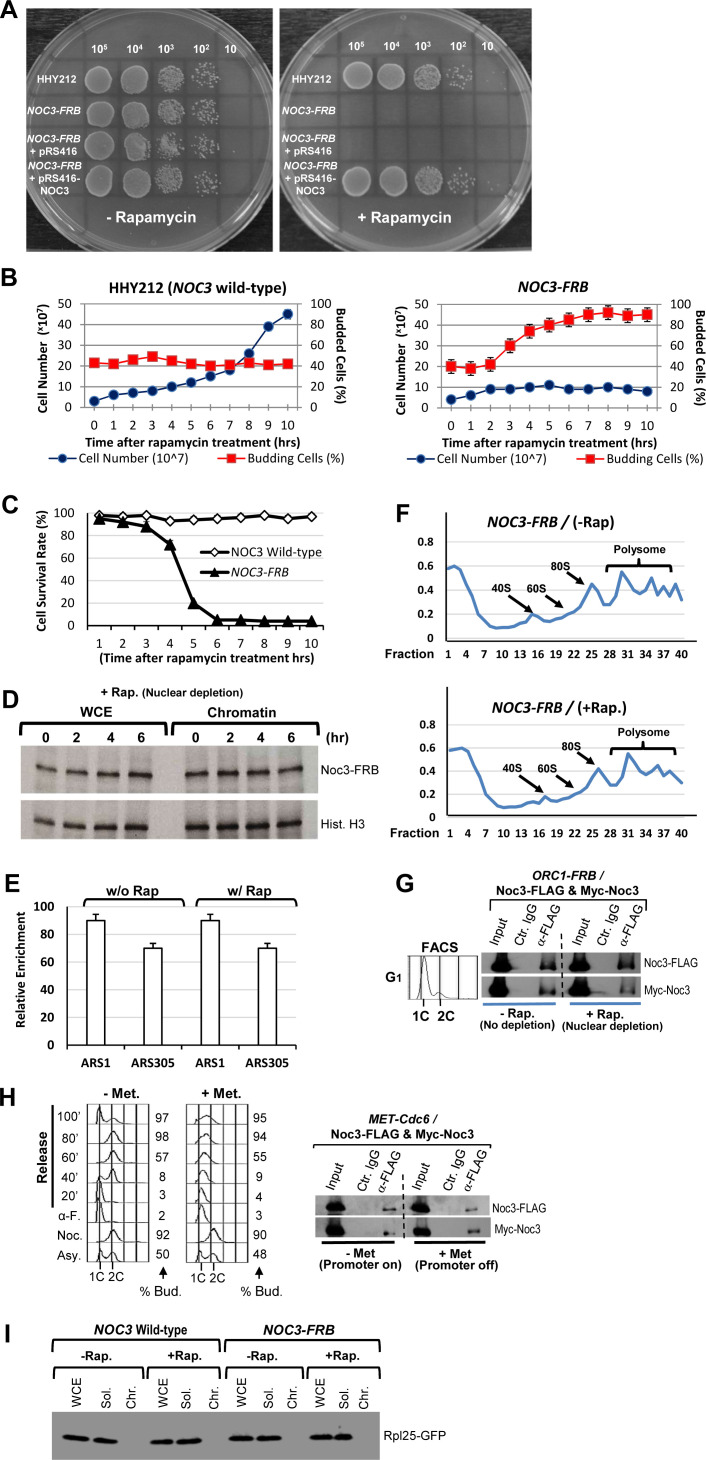
Additional data together with Fig 6 to show that depletion of non–chromatin-bound Noc3p from the nucleus during the M-to-G_1_ transition impairs Noc3p self-interaction, DNA replication, cell proliferation, and cell viability; the anchor-away system does not impair chromatin-bound Noc3p or ribosome biogenesis; the anchor-away system does not cause steric hindrance on chromatin from the ribosome; and Noc3p dimerization in G_1_-phase is independent of ORC dimerization and Cdc6p. **(A)** 10-fold serial dilutions of cells from HHY212 (NOC3 WT) and NOC3-FRB strains. The cells, containing the empty vector or a plasmid expressing the WT NOC3, were spotted on galactose-containing plates with or without rapamycin and grown at 30°C for 3 d. **(B)** HHY212 (NOC3 WT) and NOC3-FRB cells were treated with rapamycin. Cell number (circles) and percentage of budded cells (squares) were counted. **(C)** Percentages of viable HHY212 (NOC3 WT; open squares) and NOC3-FRB (closed triangles) cells were determined by plating aliquots of cells on rapamycin-free plates after the rapamycin treatment for different lengths of time. **(B, C)** Results are presented as the average ± SD of three independent experiments. **(D)** NOC3-FRB cells were harvested at the indicated time points after rapamycin was added. Whole-cell extracts and chromatin fractions were probed for NOC3-FRB and histone H3. **(E)** ChIP assays were performed using anti-FLAG antibody with YL1879 cells cultured with or without rapamycin. Real-time PCR using primers to amplify ARS1, ARS1 + 2.5 kb, ARS305, and ARS305 + 8 kb was performed. The relative enrichment (ARS normalized to non-ARS) was calculated and averaged from three independent experiments. Data are presented as the mean ± SD. **(F)** Ribosome profiles (OD260) for NOC3-FRB cells treated with or without rapamycin. Equal amounts of cell lysates were fractionated through 20–60% gradient. The 40S small subunit, 60S large subunit, 80S mono-ribosome, and poly-ribosome were indicated by arrows. **(G)** Extracts from G1-phase–synchronized ORC1-FRB cells, grown ± rapamycin, expressing Myc-Noc3 and Noc3-FLAG were immunoprecipitated with anti-FLAG antibody or control mouse IgG. Whole-cell extracts (input) and immunoprecipitates by anti-FLAG or control IgG were immunoblotted with anti-FLAG and anti-Myc antibodies. **(H)** YL1923 (MET-CDC6) cells expressing Myc-Noc3 and FLAG-Noc3 were first arrested in M-phase in nocodazole-containing medium without methionine. The culture was then split into two halves. One half was kept in methionine-dropout medium for 0.5 h and then released into α-factor–containing medium without methionine (−Met.). The other half was shifted to nocodazole- and methionine-containing medium for 0.5 h to deplete Cdc6p and then released into α-factor– and methionine-containing medium to arrest cells in G1-phase (+Met.). Flow cytometry was used to monitor cell cycle progression. Extracts prepared from aliquots of the cells were immunoprecipitated with anti-FLAG antibody or control IgG. Whole-cell extracts (input) and immunoprecipitates (IP) were immunoblotted with anti-FLAG and anti-Myc antibodies. **(I)** Whole-cell extracts, and soluble and chromatin fractions (loaded at a 1:1:5 cell equivalent ratio) of HHY212 and NOC3-FRB cells expressing Rpl25-GFP were immunoblotted with anti-GFP antibody.

In the absence of rapamycin, cells expressing *NOC3-FRB* grew well (Fig S6A), which indicates that the *NOC3-FRB* protein is functional. However, in the presence of rapamycin, the FRB-tagged Noc3p strain did not grow. The growth defects could be rescued by ectopically expressed WT Noc3p (Fig S6A). In cycling *NOC3-FRB* cells, the addition of rapamycin leads to the inhibition of cell proliferation and accumulation of budded cells (Fig S6B). Moreover, cell viability also diminished during rapamycin treatment (Fig S6C). Collectively, these results demonstrate that the depletion of free Noc3p from the nucleus impedes cell proliferation and viability.

When rapamycin was applied to growing *NOC3-FRB* cells, the chromatin-bound Noc3-FRB remained constant (Fig S6D), showing that the system did not disrupt the chromatin association of Noc3-FRB. Moreover, ChIP assay results indicate that Noc3-FRB preferentially bound *ARS1* and *ARS305*, but not the non-ARS control regions, in the presence or absence of rapamycin in *NOC3-FRB* cells arrested in M-phase by nocodazole (Fig S6E). Furthermore, normal poly-ribosome profiles were observed in both rapamycin-treated and untreated cells (Fig S6F), indicating that the anchor-away system does not interfere with ribosome biogenesis. These data demonstrate that the anchor-away system depletes free Noc3p from the nucleus without affecting ribosome biogenesis or the ARS-bound Noc3p.

**Figure 6.**
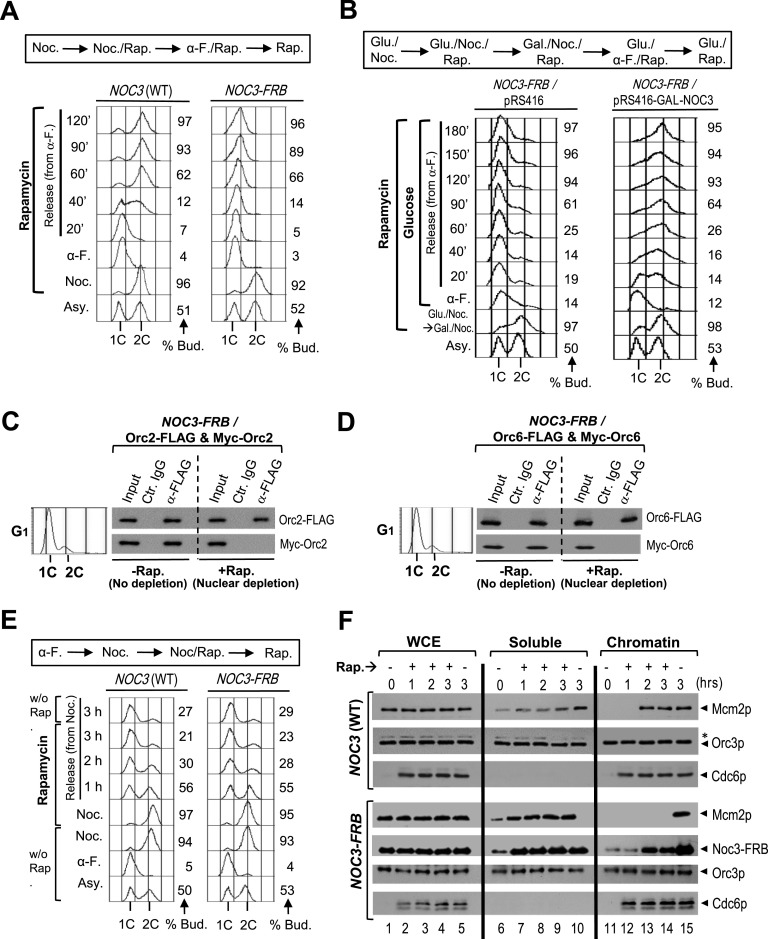
Depletion of non–chromatin-bound Noc3p by anchor-away during the M-to-G_1_ transition impedes ORC dimerization and pre-RC formation. **(A)** Rapamycin was added to nocodazole-arrested HHY212 (*NOC3* WT) and *NOC3-FRB* cells for 30 min before the cells were released into medium containing α-factor and rapamycin. The cells were then released into fresh medium containing rapamycin. Flow cytometry was performed with cell samples taken at the indicated time points. **(B)** *NOC3-FRB* cells containing pRS416 or pRS416-GAL-NOC3 plasmid were arrested in M-phase using nocodazole- and glucose-containing medium, treated with rapamycin, and then shifted to nocodazole-, galactose-, and rapamycin-containing medium. The cells were then released into α-factor-, glucose-, and rapamycin-containing medium. Lastly, the cells were released into fresh glucose- and rapamycin-containing medium. Flow cytometry and budded cell counting were performed with cells taken at the indicated time points. **(C, D)** Extracts from α-factor–arrested G_1_-phase *NOC3-FRB* cells expressing Myc-Orc2 and Orc2-FLAG (C), or Myc-Orc6 and Orc6-FLAG (D), treated or not treated with rapamycin (+/- rap.), were immunoprecipitated with anti-FLAG antibody or control mouse IgG. Whole-cell extracts (input) and immunoprecipitates by anti-FLAG antibody or control IgG were immunoblotted with anti-FLAG and anti-Myc antibodies. **(E, F)** Flow cytometry data for the experiment are shown in (F). **(F)** HHY212 (*NOC3* WT) and *NOC3-FRB* cells were synchronized in M-phase with nocodazole, and each culture was split into two halves. One half was treated with rapamycin (rap. +) for 1 h and then released into rapamycin- and α-factor–containing medium for 3 h. The other half was kept in M-phase without rapamycin for 1 h and then released into fresh medium with α-factor (without rapamycin) for 3 h, as control. Cell samples were collected at the indicated time points. Whole-cell extracts, soluble fractions, and chromatin fractions (loaded at 1:1:5 cell equivalent ratio) were immunoblotted for Mcm2p, Orc3p, Cdc6p, and Noc3-FRB; *, anti-Orc3 cross-reacting band. Note that the amount of Cdc6p in the soluble fractions was too low to be detected.

Flow cytometry was used to examine the effects of nuclear depletion of free *NOC3-FRB* after rapamycin treatment on cell cycle progression. *NOC3-FRB* cells released from the M-phase block completed M-phase and entered G_1_-phase; however, the subsequent S-phase was defective and the cells were arrested with mostly large buds and unreplicated DNA (Fig 6A). As a control, ectopically expressed Noc3p could rescue the replication defects in *NOC3-FRB* cells, showing that anchor-away per se does not produce DNA replication defects (Fig 6B). These results suggest that during the M-to-G_1_ transition, free Noc3p in the nucleus is necessary for DNA replication in the next S-phase.

ORC dimerization was then examined by co-IP with extracts expressing two differently tagged versions of the same ORC subunit from G_1_-phase–synchronized *NOC3-FRB* cells. The results show that co-IP between Orc2-FLAG and Myc-Orc2 (Fig 6C), and between Orc6-FLAG and Myc-Orc6 (Fig 6D), was observable in the absence of rapamycin (“−rap”), but not in the presence of rapamycin (“+rap”). These experiments indicate that free Noc3p is required for ORC dimerization.

On the contrary, we found that Noc3p dimerization does not require ORC dimerization (Fig S6G) or Cdc6p (Fig S6H), as positive co-IP between Noc3-FLAG and Myc-Noc3 was detectable after Orc1-FRB nuclear depletion in *ORC1-FRB* cells, and also after Cdc6p depletion in *MET-CDC6* cells, with the cells in both cases expressing Noc3-FLAG and Myc-Noc3 in G_1_-phase. Of note, Cdc6p-depleted *MET-CDC6* cells had abrogated cell cycle progression (Fig S6H), consistent with previous findings (Amin et al, 2020).

We then examined pre-RC formation after Noc3-FRB nuclear depletion in cells released from nocodazole-blocked M-phase into G_1_-phase in the presence of α-factor with or without rapamycin (Fig 6E and F). In both rapamycin-treated (Fig 6F, lanes 12–14; “+”) and untreated (Fig 6F, lane 15; “−”) *NOC3* WT control cells, Mcm2p was loaded onto chromatin after the cells were released from M-phase into G_1_-phase. The same was observed for *NOC3-FRB* cells grown in rapamycin-free medium (Fig 6F, lane 15). However, Noc3-FRB nuclear depletion by rapamycin compromised pre-RC formation in *NOC3-FRB* cells, despite Noc3-FRB and Orc3p still being present on chromatin (Fig 6F, lanes 12–14). These data strongly suggest that free Noc3p is necessary for pre-RC formation. Interestingly, Cdc6p was present on chromatin despite rapamycin treatment (Fig 6F, lanes 12–15). Noc3p is required for Cdc6p loading (Zhang et al, 2002; Huo et al, 2012). However, Noc3p dimerization is not required, as Cdc6p was loaded onto chromatin despite rapamycin treatment (Fig 6E, lanes 12–15). Therefore, the Noc3p monomer, together with the ORC single-hexamer, is sufficient for Cdc6p recruitment.

To exclude the possibility that pre-RC formation failure in the *NOC3-FRB* cells treated with rapamycin was caused by ribosomal steric interference, we show that a ribosomal protein, Rpl25p, was not present in the chromatin faction from both rapamycin-treated and untreated cells (Fig S6I), suggesting that ribosome was not even bound to the chromatin. Therefore, our data show that the DNA replication and pre-RC defects in these experiments were specific to the depletion of free Noc3p from the nucleus. Together, the results from the anchor-away (Figs 6 and S6) and *NOC3* mutant (Figs 1–3 and S1–S3) studies solidly demonstrate that Noc3p dimerizes in a cell cycle–regulated and semi-conservative manner and its dimerization is essential for ORC dimerization, pre-RC formation, and DNA replication, independent of ribosome biogenesis.

### Predicted structure models of Noc3p dimer, ORC-Noc3p monomer, and (ORC-Noc3p)_2_ dimer

**Figure S7.**
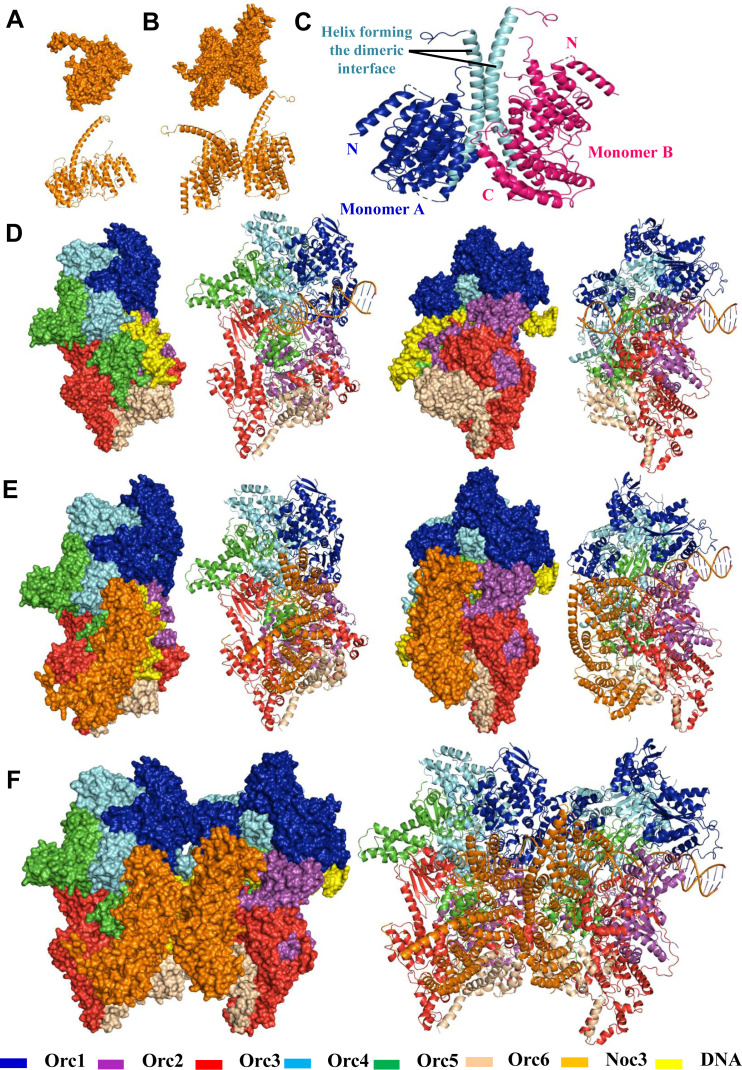
Additional data together with Fig 7 showing the Noc3p, Noc3p dimer, ORC-DNA, ORC-Noc3p-DNA, and (ORC-Noc3p)2 dimer–DNA models. **(A, B, C, D, E, F)** Modeling was performed manually (B, D, E, F) or by using HADDOCK2.2 (C), with the published Noc3p (A) and ORC (D) cryo-EM structures as inputs. Ribbon diagrams and space-filling models are provided. **(D, E)** Views from two angles are shown in (D, E). **(A)** Published Noc3p monomer structure. **(B)** Noc3p dimer model. **(C)** Noc3p dimer model built by using HADDOCK2.2. Our model indicates that Noc3p can form a dimer through the central helix. Noc3p is composed of a helix bundle in which a long and central helix is formed by residues 370–420 aa. The terminals, monomers, and structures are as indicated. **(D)** Published model of ORC single-hexamer with DNA. **(E)** Model of ORC single-hexamer with Noc3p and DNA. **(F)** Model of (ORC-Noc3p)2 dimer with DNA.

To provide structural support for Noc3p dimerization, we performed modeling of the Noc3p dimer. The cryogenic electron microscopy structure of Noc3p (aa 271–430, PDB code: 6EM5; Kater et al, 2017) was used as the input (Fig S7A). The Noc3p dimer structure model was built manually (Fig S7B) or by using HADDOCK2.2 (Dominguez et al, 2003) (Fig S7C). Noc3p is composed of a helix bundle in which a long and central helix is formed by the residues aa 370–420 (included in the F7 fragment; aa 326–512; Fig S4). Our model indicates that Noc3p can form a dimer through the central helix, consistent with our conclusion that Noc3p dimerizes. Using the published ORC single-hexamer cryo-EM structure with DNA (Fig S7D; PDB code: 5ZR1; Li et al, 2018), we generated the structure model of ORC single-hexamer binding with Noc3p (Fig S7E, two different planes of view have been provided). Consistent with previous reports (Zhang et al, 2002; Huo et al, 2012; Wu et al, 2019), the model indicates that Noc3p interacts with ORC2, ORC3, ORC5, and ORC6, whereas both Noc3p and ORC have interactions with DNA without any steric hindrance. Modeling was also performed for the (Noc3p-ORC)_2_ dimer structure (Fig S7F). Also consistent with our previous findings (Amin et al, 2020), our (Noc3p-ORC)_2_ dimer model indicates that all ORC components interact with each other, whereas each ORC single-hexamer maintains interactions with a Noc3p in the Noc3p-Noc3p dimer through Noc3p’s interactions with ORC2, ORC3, ORC5, and ORC6. ORC and Noc3p also maintain their interactions with DNA in this model without steric impediments.

## Discussion

### Separation-of-function mutants of *NOC3* in ribosome biogenesis and DNA replication

Substantial data from the plasmid loss, nuclear Rpl25-GFP localization, dosage lethality, ribosome profile, co-IP, re-ChIP, sucrose gradient centrifugation, chromatin-binding, and flow cytometry assays with WT cells and different separation-of-function *NOC3* mutants strongly support our previous findings that *NOC3* plays multiple, yet separable, essential roles in both ribosome biogenesis and DNA replication. The phenotypes of various *NOC3* mutants demonstrate that ORC dimerization, pre-RC formation, and DNA replication are impaired in cells harboring replication-defective *NOC3* mutant alleles, but not those (such as *noc3-1-ribo*) with ribosome biogenesis deficiency only. We also established that the DNA replication defects did not result from defects in ribosome biogenesis, and vice versa, in the *NOC3* mutants. Collectively, our *NOC3* mutant studies confirm that Noc3p plays a direct role in ORC dimerization, pre-RC formation, and DNA replication, while serving another function in ribosome biogenesis in vivo. This is the first time since the discovery of Noc3p as a novel replication-initiation protein (Zhang et al, 2002) that the molecular mechanism of Noc3p as an essential mediator of ORC dimerization in pre-RC formation in vivo has been elucidated.

Interestingly, MCM single-hexamers were also absent in the sucrose gradient experiments without functional Noc3p. This is consistent with our previously published data showing that MCM single-hexamers were absent when pre-RC formation failed in G_1_-phase cells depleted of Cdt1p (Wu et al, 2012) or Cdc6p (Amin et al, 2020). It is likely that non–chromatin-bound MCM single-hexamers are unstable.

Furthermore, our chromatin-binding, co-IP, and re-ChIP assays demonstrate that Noc3p self-interacts and dimerizes in a cell cycle–regulated manner and that two molecules of Noc3p co-occupy the same origins of replication in an early step of pre-RC formation. The specific self-interactions of Noc3p in G_1_-phase, but not in S- or M-phase, strongly suggest that these interactions were not artifacts in the experiments. Using various *NOC3* domains and domain-deletion mutants, the Noc3p-self-interacting coiled-coil domain was also shown to be essential for Noc3p dimerization and cell proliferation.

Significantly, our data also indicate that free Noc3p binds to chromatin-bound Noc3p to form dimers in late M-phase before MCM loading, and Noc3p de-dimerizes in the S-phase. Similar findings were previously reported by us for ORC (Amin et al, 2020). Critically, depleting non–chromatin-bound Noc3p using the anchor-away system to prevent Noc3p dimerization abolishes pre-RC formation, DNA replication, cell proliferation, and cell viability. To corroborate our in vivo findings in this study, structural modeling of the Noc3p dimer, ORC-Noc3p monomer, and (ORC-Noc3p)_2_ dimer suggests that Noc3p forms a dimer through the central helix (which contains the Noc3p-self-interacting coiled-coil domain) and that this structure interacts with the ORC dimer and DNA.

**Figure 7.**
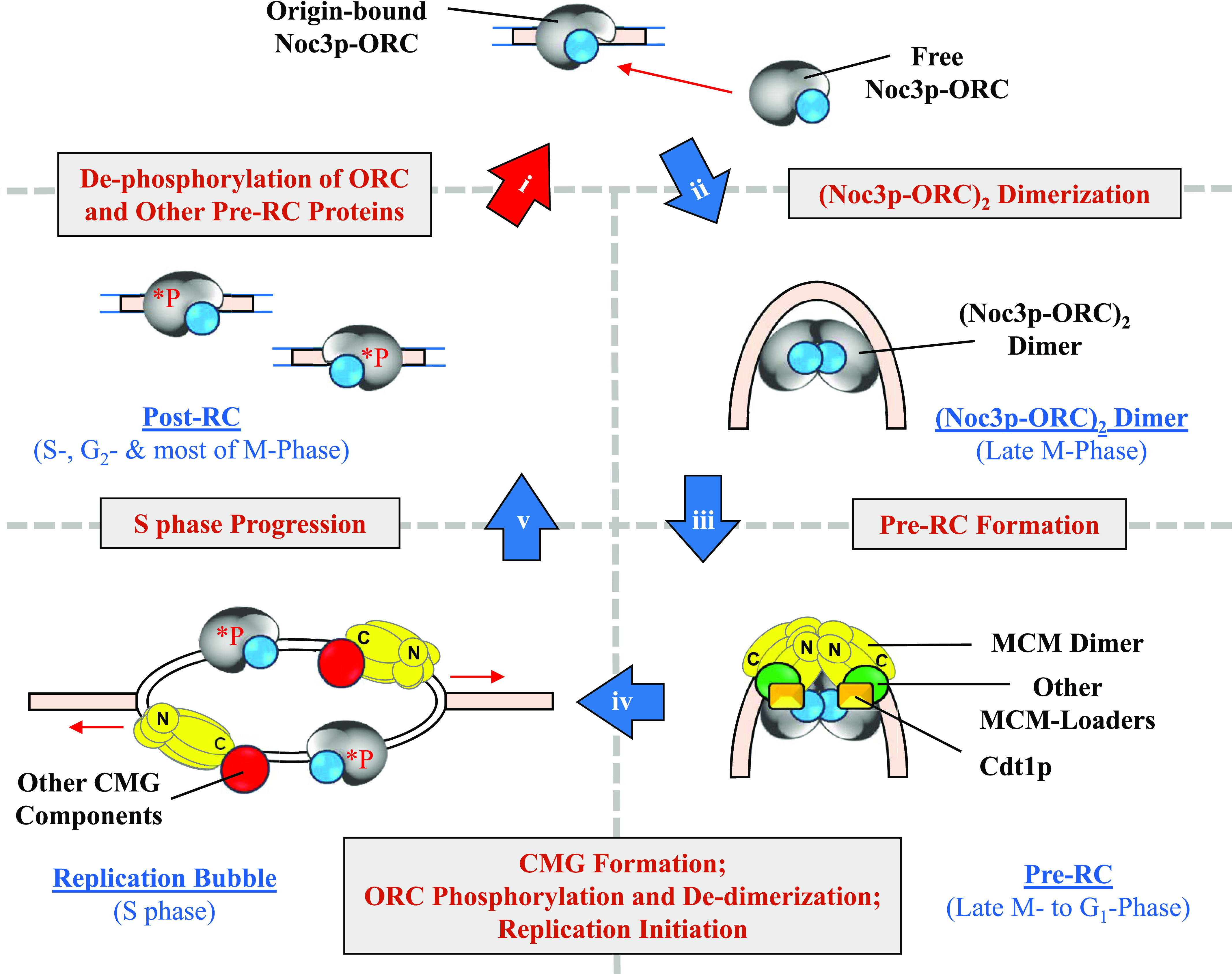
Models for (Noc3p-ORC)_2_ dimerization, pre-RC formation, and (Noc3p-ORC)_2_ de-dimerization. Free (non–chromatin-bound) Noc3p-ORC binds to the origin-bound Noc3p-ORC at each replication origin by protein–protein interactions to form a (Noc3p-ORC)_2_ dimer at the M-to-G_1_ transition to enable loading of the MCM double-hexamer. Upon replication initiation, each (Noc3p-ORC)_2_ dimer de-dimerizes and separates into two Noc3p-ORCs, which bind and protect the two nascent replication origins at each replication bubble (See Discussion section for details).

The dimerization cycle of Noc3p supported by our results, combined with our ORC dimerization model (Amin et al, 2020), signifies a novel model for pre-RC assembly (Fig 7). At the M-to-G_1_ transition, ORC is de-phosphorylated by Cdc14p (Zhai et al, 2010) and bound to replication origins together with Noc3p (Zhang et al, 2002) as a Noc3p-ORC, or “monomer” (Fig 7, Step i). Before MCM loading, free ORC and Noc3p bind to the origin-bound Noc3p-ORC at each replication origin by protein–protein interactions to form a (Noc3p-ORC)_2_ dimer, providing a symmetric platform for symmetric pre-RC formation (Fig 7, Step ii). This process is mediated by Noc3p-Noc3p dimerization. Based on the finding that when two ORCs bind to the same *ARS1*, one binds at ACS and the other at B2 (Miller et al, 2019), we propose that after (Noc3p-ORC)_2_ dimerization, the two Noc3p-ORCs in a (Noc3p-ORC)_2_ dimer occupy two separate areas of an origin, possibly the ACS and B2 of *ARS1*, leaving a space in-between for MCM loading (Fig 7, Step ii). The (Noc3p-ORC)_2_ dimer bends the origin DNA (Lee & Bell, 1997; Sun et al, 2012; Li et al, 2018; Amin et al, 2020) to facilitate the loading of the MCM double-hexamers. With the help of other MCM-loading factors, such as Ipi3p (Huo et al, 2012; Huang et al, 2016a) and Cdc6p, the symmetric (Noc3p-ORC)_2_ dimer simultaneously and synergistically loads the symmetric MCM double-hexamer associated with two Cdt1p molecules that bridge between Noc3p-ORC and MCM proteins, forming pre-RC (Fig 7A, Step iii). In principle, this mechanism is more efficient than sequential MCM loading. Upon CMG formation, ORC phosphorylation, and replication initiation, each (Noc3p-ORC)_2_ dimer de-dimerizes and separates into two Noc3p-ORCs, which bind and protect the two nascent replication origins at each replication bubble (Fig 7A, Step iv). This resolved an important issue that had not been previously addressed. The active CMG complexes move away from replication origins during S-phase progression and then disassociate from chromatin (Fig 7, Step iv). In this model, the (ORC-Noc3p)_2_ dimer at each licensed origin is formed by one Noc3p-ORC from the parental chromosome, and one Noc3p-ORC newly recruited in late M-phase. This semi-conservative dimerization cycle ensures that replication origins are continuously protected by Noc3p-ORC to prevent invasion by nucleosomes and/or other proteins, thereby safeguarding faithful genome duplication (see Introduction section).

Consistent with previous models, our model depicts that ORC interacts with the C-terminal region of MCM2-7p via Cdt1p, and Mcm2-7p self-interacts via the N-terminal regions in the MCM double-hexamer (Coster & Diffley, 2017; Amin et al, 2020). Of note, previous studies have shown that Cdt1p interacts with Mcm6p (Wu et al, 2012, 2019) and that Noc3p also interacts with both Mcm6p and Cdt1p (Huo et al, 2012; Wu et al, 2019). In our models, the interactions of Noc3p with ORC, Cdt1p, and MCM are accommodated. Our results (Figs 4 and S4) and predictive structural modeling data (Fig S7) indicate that Noc3p self-interaction occurs at the C-terminal CC2 domain, whereas Noc3p also interacts with Orc2, Orc3, Orc5, and Orc6. The predictive structural models suggest that Noc3p interacts with ORC via its N-terminal domain (Fig S7D and E). The models shown in Fig S7 also take into consideration of previous reports suggesting that DNA unwinds at the N-terminus of MCM (Coster & Diffley, 2017; Georgescu et al, 2017) and that the Mcm2-7p hexamers pass each other after pre-RC conversion into CMG (Cdc45p-MCM-GINS; Ticau et al, 2015; Coster & Diffley, 2017). The models proposed in the existing literature do not resolve how MCM double-hexamers separate into monomers or how the MCM monomers circumvent the origin-bound ORC roadblock, although it has been suggested that a “sliding helicase-loading intermediate” may resolve this issue (Warner et al, 2017). It has been suggested that ORC changes configuration or transiently dissociates from the origins to resolve this issue (Warner et al, 2017). A more plausible solution is offered by the (Noc3p-ORC)_2_ dimerization model, which necessitates that after de-dimerization, each of the two new nascent origins is bound by one Noc3p-ORC and one MCM single-hexamer (in CMG), allowing for bi-directional movement of MCM without being blocked by Noc3p-ORC (Fig 7, Step iv).

### Previous evidence for Noc3p playing separate functions in DNA replication and ribosome biogenesis

Earlier studies established that Noc3p plays essential roles in cell growth, development, differentiation, and apoptosis (Massari & Murre, 2000; Robinson & Lopes, 2000). A later study demonstrated that Noc3p is also required for pre-ribosome maturation and pre-rRNA processing (Milkereit et al, 2001). Screening of a yeast genomic DNA library in a subsequent study identified Noc3p having a role in DNA replication licensing (Zhang et al, 2002). Similar to many other pre-RC proteins, Noc3p forms complexes with ORC and MCM through direct physical protein–protein interactions, binds specific ARSs on chromatin in vivo, and is required for the recruitment of CDC6p and MCM proteins for pre-RC formation, and for the maintenance of MCM proteins on chromatin in G_1_-phase cells (Zhang et al, 2002; Huo et al, 2012). Noc3p plays a direct role in DNA replication, and it was ruled out that the role of Noc3p in DNA replication might be an indirect result of ribosome deficiency in the *NOC3* mutants and that Noc3p might affect the transcription of other replication-initiating factors (Zhang et al, 2002; Huo et al, 2012). Significantly, the cellular levels of known pre-RC proteins were not reduced within the pre-RC formation time window when Noc3p was depleted in yeast or human cells (Zhang et al, 2002; Huo et al, 2012; Cheung, 2019), and the overexpression of the stable, yet functional, Cdc6-NΔ did not rescue pre-RC formation (Zhang et al, 2002). Furthermore, the overexpression of Noc3p driven by the *GAL* promoter leads to dosage lethality in ORC and Cdc6p mutant yeast cells, similar to that observed for the overexpression of MCM and other DNA replication-initiation proteins (Honey & Futcher, 2007; Zhai et al, 2011). Reciprocally, the overexpression of pre-RC proteins also leads to dosage lethality/sickness in the replication-deficient, but not replication-proficient, *NOC3* mutants (this study). These genetic interactions strongly support the physical and functional interactions between Noc3p and other pre-RC proteins.

Previous studies suggested that the human homolog of Noc3p (FAD24/hNOC3) is involved in DNA replication and cell differentiation (Tominaga et al, 2004; Johmura et al, 2008a). Similar findings were reported in mice (Johmura et al, 2008b). A subsequent study established that hNOC3 also plays an essential role in pre-RC formation, independent of its role in ribosome biogenesis (Amin et al, 2019; Cheung et al, 2019). As found in budding yeast, hNOC3 has direct physical protein–protein interactions with several hORC and hMCM subunits and binds chromatin, preferentially associating with known origins of replication (Cheung et al, 2019). The silencing of hNOC3 impaired the chromatin association between hCDC6 and hMCM proteins, thus impeding pre-RC formation and DNA replication (Cheung et al, 2019). Importantly, inhibiting ribosome biogenesis by silencing Pol I using siRNA or preventing rRNA synthesis with a Pol I inhibitor did not prevent pre-RC formation within the experimental time window. Furthermore, Noc3p chromatin association stabilizes the pre-RCs in both budding yeast and human G_1_-phase cells (Zhang et al, 2002; Cheung et al, 2019). These findings suggest that the role of Noc3p in DNA replication is conserved between budding yeast and humans.

The physical and genetic interactions of Noc3p with so many pre-RC proteins, chromatin, and replication origins (Zhang et al, 2002; Huo et al, 2012; Wu et al, 2019) make it impossible that these interactions could be related to ribosome biogenesis rather than DNA replication. On the contrary, several other DNA replication-initiation proteins such as ORC, Cdc6p, Ipi3p, and MCM have been reported to have roles outside of DNA replication, in processes such as transcriptional silencing (Foss et al, 1993; Loo et al, 1995), mitosis control (Bueno & Russel, 1992; Calzada et al, 2001; Weinreich et al, 2001), transcription (Yankulov et al, 1999), and ribosome biogenesis (Huo et al, 2012; Huang et al, 2016b). Other ribosome biogenesis proteins, such as Yph1p (Du & Stillman, 2002), have also been shown to have roles in DNA replication. Some of these proteins may serve to coordinate ribosome biogenesis with DNA replication by participating in both processes, possible through certain differential regulatory mechanisms. For example, Noc3p forms a heterodimer with Noc2p in ribosome biogenesis (Milkereit et al, 2001), and yet forms a homodimer for replication licensing as reported here.

### Current models of replication licensing

Despite there being ample evidence to suggest that additional proteins are required for pre-RC formation, previous studies have primarily focused on the most studied pre-RC proteins (Orc1-6p, Cdc6p, Cdt1p, and Mcm2-7p) (Sun et al, 2012, 2013, 2014; Li et al, 2015; Ticau et al, 2015; Coster & Diffley, 2017; Yuan et al, 2017; Zhai et al, 2017; Miller et al, 2019). Interestingly, different pre-RC formation models have been proposed. It is not inconceivable that this may result from the differences in the in vitro experimental conditions, which likely favor the formation of certain structures over others.

The “one-ORC” model proposes that an asymmetric ORC structure loads the symmetric MCM dimer (Takara & Bell, 2011; Fernández-Cid et al, 2013; Sun et al, 2013; Yardimci & Walter, 2014; Li et al, 2015; Ticau et al, 2015; Bell & Labib, 2016; Coster & Diffley, 2017; Warner et al, 2017; Zhai et al, 2017). The “two-ORC” model proposes “quasi-symmetrical” loading of MCM by two ORCs at different replication origins (Coster & Diffley, 2017). Although their results are consistent with two ORCs synergistically loading two MCM single-hexamers, conceivably through ORC-ORC interactions based on our discovery (Amin et al, 2020), the authors did not present an ORC dimerization model.

A recent study has attempted to reconcile the two contentious models; however, the conclusions still rely primarily on evidence from in vitro experiments, in which a variety of structures and orientations of ORC and MCM bound on the origin DNA were found (Miller et al, 2019). Although the origin DNA bound by one ORC was overwhelmingly prevalent (70% at the A element and 4% at the B2 element), two inverted ORCs (18%; the MCM-interacting domains face each other) and tandem ORCs (8%; B2 element–bound ORC is facing the same direction as the A element–bound ORC) at the same origin were also found.

Interestingly, ORC double-hexamers (<10% of the purified ORC) have also been previously detected by cryo-EM (Sun et al, 2012). Furthermore, in vitro single-molecule fluorescence studies observed DNA-bound ORC double-hexamers (5% of the population) during Mcm2-7p association (Ticau et al, 2015). These data are consistent with ORC’s ability to dimerize. The low abundance of ORC dimers in these experiments is attributable to the low affinity of purified ORC for self-interactions in an in vitro environment, without the Noc3p mediator as reported in this study. It can also be reasoned that the specific conditions of in vitro experiments, which did not include all factors involved in replication licensing, may produce different results and conclusions that may represent different aspects, rather than a comprehensive view of the pre-RC formation process in the cell.

Importantly, we found that free ORC proteins do not associate with DNA during S-, G_2_-, and most of M-phase; that is, there is no net increase in ORC proteins on the chromosomes as they are duplicated (Amin et al, 2020; this study). Therefore, the “one-ORC” and “two-ORC” models cannot address the issue of how newly replicated replication origins can be protected by ORC from potential invasion by histones and other proteins. On the contrary, our semi-conservative (Noc3p-ORC)_2_ dimerization model may be able to resolve this issue, as it depicts that each nascent origin can be protected by a single Noc3p-ORC separated from a (Noc3p-ORC)_2_ dimer upon replication initiation.

### New insight into the “(Noc3p-ORC)_2_ dimerization cycle”

Both Noc3p and ORC interact, and both are continuously bound to the chromatin throughout the cell cycle (Zhang et al, 2002; Huo et al, 2012; Amin et al, 2019; Cheung et al, 2019; Amin et al, 2020). This is probably achieved by Noc3p’s interactions with Orc2, Orc3, Orc5, and Orc6 (Zhang et al, 2002; Huo et al, 2012), which form the lower half (over 2/3) of the Orc1-6p single-hexamer structure. Like Orc1-6p, Noc3p also has self-interactions (Wu et al, 2019; this study). Interestingly, like yeast Noc3p and ORC, hORC and hNOC3 also self-interact (Wu et al, 2019). Although this aspect of our work requires further development, it supports an essential and cell cycle–regulated (Noc3p-ORC)_2_ dimerization model that is conserved in eukaryotes. Although it has been speculated that the ORC dimerization model may provide a solution to the problem of replication origin inheritance to daughter cells in higher eukaryotes (Amin et al, 2020), as the sequence dependence of ORC binding in higher eukaryotes is not as strict as in budding yeasts, Noc3p may serve to enforce more stringent regulation on origin selection, as two ORC-Noc3p complexes split from a (Noc3p-ORC)_2_ dimer may potentially occupy and mark the two sister origins after DNA replication initiation. Our findings shed new light upon the proposed “ORC dimerization cycle” in eukaryotes (Amin et al, 2020) that ensures stringent once-per-cell-cycle DNA replication.

We propose a novel form of the “ORC dimerization cycle” based on our data and analysis of existing publications: the cell cycle–dependent and likely semi-conservative ORC dimerization cycle (Amin et al, 2020) mediated by Noc3p dimerization. This process is essential for replication licensing and the regulation of DNA replication. Our model complements the existing models and presents a more comprehensive insight into the regulatory mechanisms of (Noc3p-ORC)_2_ dimerization in pre-RC formation and DNA replication. Further studies into the mechanisms that control the (Noc3p-ORC)_2_ dimerization cycle are warranted.

An implication from this study is that Noc3p performs separate functions in DNA replication and ribosome biogenesis by forming different protein complexes; that is, Noc3p-Noc3p homodimers are involved in DNA replication (this study), whereas Noc2p-Noc3p heterodimers play roles in ribosome biogenesis as reported (Milkereit et al, 2001). This may lead to further studies to elucidate the roles of Noc3p and related proteins in coordinating the two fundamental processes in cell proliferation, namely, DNA replication and ribosome biogenesis.

## Materials and Methods

### Budding yeast strains and construction

The budding yeast, W303-1A (WT) strain, its modified strains, and strains from previous publications were used in this study. The source and genotypes of these strains have been stated in the key resources table (Table S1). Strains that were generated in this study are listed in Table S2. The FRB strains used in this study were generated in the HHY212 yeast strain background as previously described (Haruki et al, 2008). The deletion and tagging of genes was performed using PCR-based techniques (Longtine et al, 1998).

Table S1 Key resources table.

Table S2 Yeast strains, related to methods.

### Generation of temperature-sensitive (ts) mutants of *NOC3* by random mutagenesis and of *noc3△CT* by site-directed mutagenesis

The endogenous promoter of *NOC3* on the chromosome was replaced with the galactose-inducible promoter (*pGAL1-10*) by homologous recombination (one-step integration)r. The expression of *GAL-NOC3* can be suppressed by glucose (Fig S1A). *GAL-NOC3* cells cannot grow on glucose-containing media and were used for the screening and testing of *NOC3* mutants.

Random mutagenesis of *NOC3* was performed as previously described for *ORC6* (Amin et al, 2020). WT *NOC3* with its own promoter and terminator was cloned into the pRS414 vector (pRS414-NOC3). A pair of primers (pRS414 F: TGAGCGCGCGTAATACGACTC; pRS414 R: GCTTCCGGCTCCTATGTTGTG) from the multiple cloning site were used to amplify the *NOC3* gene for random mutagenesis by error-prone PCR. The PCR product and the pRS414 vector linearized by *EcoR*I and *BamH*I double digestion were gel-purified and were used to co-transform *GAL-NOC3* cells. After transformation, cells were cultured in YPG medium for 6 h to allow homologous recombination to form plasmids. The transformants were then spread on YPD plates to obtain 100–200 colonies per plate.

Replica plating was carried out after the colonies had grown to a proper size. The duplicated plates were incubated at 25°C or 37°C. The replicated plates at 37°C were compared with the plates at 25°C to identify temperature-sensitive (ts) mutant candidates that grew at 25°C but not 37°C. To confirm that the ts phenotypes resulted from mutations in *NOC3*, the candidates from the primary screen were streaked on YPD and YPG at 37°C. Mutants that could be rescued by the galactose-induced expression of WT Noc3p from *GAL-NOC3* were identified as likely *NOC3* ts mutants. The plasmids from the candidates were recovered and used to re-transform *GAL-NOC3* cells to confirm the ts phenotypes. After screening around 100,000 transformants, some candidates were identified as ts mutants. The plasmids containing the *NOC3* ts mutant genes were sequenced to identify the mutation sites (*noc3-9*, G100E, GGA to GAA; *noc3-142*, A142G, GCC to GGC).

A *NOC3* C-terminal truncation mutant (*noc3*△*CT*) at its endogenous locus was constructed using the standard PCR-based site-directed mutagenesis method as previously described (Toulmay & Schneiter, 2006). A PCR fragment containing the mutated *NOC3* (at the 1847^th^ and 1848^th^ nucleotide T and C to A and G, creating a stop codon, AGA) and a selectable marker was obtained. This PCR fragment was used to transform W303-1a cells. By homologous recombination, the mutant fragment was inserted at the WT *NOC3* locus, resulting in the replacement of WT *NOC3* by *noc3*△*CT*, which was confirmed by PCR and sequencing. The *noc3*△CT mutant was ts and subsequently re-named *noc3-3*. All *NOC3* ts mutants were integrated into the W303-1a strain, replacing the endogens *NOC3*.

### Yeast culture

Standard methods were used to grow *Saccharomyces cerevisiae* cells (Zhang et al, 2002; Wu et al, 2019; Amin et al, 2020). Cells were streaked or spread on plates and grown in incubators. Liquid cell cultures were grown in an orbital shaker and incubator (Gallenkamp) at 200–250 rpm. W303-1A cells were grown at 30°C or otherwise noted. For ts mutants, 25°C was the permissive temperature, whereas 37°C was the non-permissive/restrictive temperature. In most of the experiments, YPD complete medium (1% yeast extract, 2% peptone, and 2% glucose in distilled water) was used for cell growth. SCM-minus media lacking one or several essential amino acids and/or nucleotides were used to select transformants containing plasmids (Table S3) expressing selective markers.

Table S3 Plasmids for yeast cell transformation, related to methods.

### Yeast two-hybrid assay

The Matchmaker system III (Clontech) was used as previously described (Kan et al, 2008; Wang et al, 2010; Wu et al, 2019; Amin et al, 2020). Interactions between AD- and BD-fusion proteins were examined on SCM-Trp-Leu (synthetic complete medium, lacking tryptophan and leucine; selective for the two vectors but non-selective for the reporter gene), SCM-Trp-Leu-His (lacking tryptophan, leucine, and histidine; selective for the *HIS3* reporter gene), and SCM-Trp-Leu-His-Ade (lacking tryptophan, leucine, histidine, and adenine; selective for the *HIS3* and *ADE2* reporter genes) plates.

As an example, the *noc3-CC2Δ* mutant failed to interact with Noc3p; however, its interactions with Orc2p and Orc3p were still maintained (Fig S4G), and these results were also corroborated by the co-IP assays shown in Fig 4E and F. These findings indicate that the results of the yeast two-hybrid assays were not a consequence of indirect protein–protein interactions. Of note, indirect interactions mediated by other proteins in the host yeast cells are mostly undetectable in our study as evidenced by the lack of false-positive results in our study. This is all the more plausible considering that pre-RC proteins typically form large complexes.

### Cell cycle synchronization and flow cytometry

Budding yeast cell cycle synchronization and release with 5 µg/ml α-factor (G_1_-phase), 150 mM hydroxyurea (S-phase), or 10–20 µg/ml nocodazole (M-phase) were carried out as previously described (Wu et al, 2012; Amin et al, 2020). Flow cytometry was also performed as previously described (Zhang et al, 2002; Amin et al, 2020).

### Chromatin-binding assay

To determine the chromatin association patterns of pre-RC proteins throughout the cell cycle, chromatin-binding assays were performed as described in previous publications (Wu et al, 2012; Amin et al, 2020).

For the chromatin-binding experiments with cells harvested at different time points after they were synchronized and released, the relative levels of endogenous Noc3-HA (where applicable), Orc3p, Mcm2p, and histone H3 (for the chromatin fractions)/β-actin (for the soluble protein fractions) were determined.

For the chromatin-binding experiments shown in Fig 5C, pESC-Myc-Noc3–expressing cells were synchronized in G_1_-phase in glucose and α-factor–containing medium to suppress the expression of Myc-Noc3. The cells were subsequently induced to express Myc-Noc3 in galactose- and α-factor–containing medium before being released into glucose-containing medium (to suppress Myc-Noc3 expression) for cell cycle progression. Whole-cell extracts and chromatin-bound proteins from cells harvested at the indicated time points were immunoblotted to detect Myc-Noc3, Mcm2p, and histone H3. DNase I–solubilized chromatin fractions were analyzed in this experiment instead of crude chromatin fractions, as ectopically expressed Myc-Noc3 may bind to non-chromatin materials in the crude chromatin preparations.

### Sucrose sedimentation gradient analysis

Sucrose sedimentation gradient analysis was done as previously described (Chen et al, 2011; Wu et al, 2012; Amin et al, 2020). The experimental conditions that are related to Noc3p depletion are described in the Results section and figure legends.

For the experiments shown in Figs 3H and I, S3A–J, and S5C–E, we reasoned that the small amount of ORC appearing as small complexes (single-hexamers) in G_1_-phase cells likely resulted from the slight asynchrony in the cell population and/or partial disruption of the ORC double-hexamers by the harsh experimental procedures involving the use of SDS, which was essential for solubilizing ORC.

### Co-IP assays

Co-IP assays were performed as previously described (Huo et al, 2012; Wu et al, 2012; Amin et al, 2020). The experimental conditions that are related to Noc3p depletion are described in the Results section and figure legends for the experiments shown in Fig 3A–F.

We optimized the conditions for the *GAL* promoter–driven ectopic expression of the tagged ORC proteins as reported in our previous work (Amin et al, 2020), to ensure that the expression levels of the ectopically expressed ORC were similar to the endogenous ORC in co-IP and all other experiments. Cell extracts were also DNase I–digested to exclude the possibility of DNA-mediated indirect interactions. The expression of ectopic protein was induced for 1 h under the appropriate conditions (Amin et al, 2020).

### Sequential chromatin immunoprecipitation (re-ChIP) assay

Sequential chromatin immunoprecipitation (re-ChIP) was carried out as previously described (Geisberg & Struhl, 2004; Huo et al, 2012; Amin et al, 2020) with minor modifications. For the experiments shown in Fig 3G, sequential ChIP (re-ChIP) assays were performed with G_1_-synchronized *noc3-1-ribo* or *noc3-3-rep* cells co-expressing Orc6-FLAG and Myc-Orc6 shifted to 37°C, whereas W303-1A cell extracts co-expressing Noc3-FLAG and Myc-Noc3 were used in the experiments shown in Fig 4C.

The primers used for PCR amplification were ARS1: ARS1 1 F 5′-GAAATAGGTTATTACTGAGTAG-3′ and ARS1 1 R 5′-CCTGCGATGTATATTTTCCTG-3′; ARS1 + 2.5 kb: Con R2.5 F 5-′CATCAATTGTGCACTCGGAC-3′ and Con R2.5 R 5′-GAACACGGCAATTGTAGGTGG-3′; ARS501: ARS 501 F 5′-CTTTTTTAATGAAGATGACATTGCTCC-3′ and ARS 501 R 5′-GATGATGATGAGGAGCTCCAATC-3′; ARS501 + 11 kb: Con 501 F 5′-CACCGATACGTACTTAAACTCTTCCG-3′ and Con 501 R 5′-GAGAAAGCTTAGTCCATTCGGCC-3′; ARS305: ARS 305 F 5 -′CTCCGTTTTTAGCCCCCCGTG-3′ and ARS 305 R 5′-GATTGAGGCCACAGCAAGACC-3′; and ARS305 + 8 kb: Con 305 F 5′-GGTGGTGGAGAAGCGGTTCAAAG-3′ and Con 305 R 5′-CCGCTCGTACCCGCTCCTGA-3′.

ORC proteins in G_1_-phase cells cannot be efficiently extracted using regular protein extraction techniques (even with DNase I digestion), as they tightly bound to the nuclear matrix (Huo et al, 2012; Amin et al, 2020). We therefore followed the modified method (Huo et al, 2012; Amin et al, 2020) by cross-linking the cells using formaldehyde and then solubilizing ORC from the crude chromatin fractions using DNase I digestion and subsequent extraction with SDS. These extracts were then diluted (to reduce the SDS concentration) before being used for ChIP and re-ChIP assays. Application of these stringent conditions also reduces the likelihood of non-specific protein–DNA and protein–protein interactions (Amin et al, 2020).

### Anchor-away system

Experiments using the anchor-away system (Haruki et al, 2008) were performed as described previously (Amin et al, 2020).

### Quantitative plasmid loss assay

Quantitative plasmid loss assay was performed as described previously (Zhang et al, 2002; Ma et al, 2010; Wu et al, 2012).

### Fluorescence microscopy of Rlp25-GFP

Fluorescence microscopy of Rlp25-GFP was carried out as previously described (Wu et al, 2012).

### Computation prediction of coiled-coil motifs and dimeric–trimeric coiled coils

The coiled-coil motifs were predicted by the COILS server with a 28-aa window (Lupas et al, 1991). The MultiCoil program was used to predict the dimeric–trimeric coiled-coil regions with a 28-aa window (Wolf et al, 1997).

### Multiple sequence alignments

DNA sequence homology was examined in terms of percentage, using the ClustalW2 online tool from EMBL-EBI (http://www.ebi.ac.uk/) (EMBL-EBI, 2020). This program aligned the self-interacting domain sequences to produce an alignment profile and a summary table displaying sequence homology within all the selected species (EMBL-EBI, 2020).

### Noc3p dimer, ORC-Noc3p monomer, and (ORC-Noc3p)_2_ dimer structure modeling

A Noc3p dimer model (Fig S7B) was manually built based on the published cryo-EM structure of Noc3p (Fig S7A; aa 271–430, PDB code: 6EM5; Kater et al, 2017); Noc3p dimer modeling was also carried out using HADDOCK2.2 (Fig S7C) in accordance with published protocols (Dominguez et al, 2003). The cryo-EM structure data of Noc3p were used as the input, and residues 360–440aa, in which the residues are exposed to solvent, were defined as active residues and used as ambiguous interaction restraints. The simulations were run using the standard HADDOCK protocol with 1,000 initial docking simulations followed by 200 refinement simulations and subsequently 200 final refinement simulations including explicit water molecules. The final clustering was done based on the RMSD value using 7.5 Å as the threshold. The ORC single-hexamer with Noc3p and DNA model (Fig S7E) was manually built using the published cryo-EM structure of ORC single-hexamer with DNA (Fig S7D; PDB code: 5ZR1; Li et al, 2018) and interaction data from previous publications (Zhang et al, 2002; Huo et al, 2012; Wu et al, 2019). The (ORC-Noc3p)_2_ dimer model (Fig S7F) was manually built based on the aforementioned data.

### Quantification and statistical analysis

ImageJ (Fiji) was used to process all acquired raw immunoblot images. Quantifications of the protein signal intensities in the chromatin fractions shown in Figs 2C and D, S2C–E, 5B, and S5B are presented as the average ± SD of three independent experiments. For these experiments, the signals of Orc3p and Mcm2p and/or Noc3-HA were normalized to that of histone H3 at different time points, and the resulting numbers were then further normalized to the G_1_-phase sample (αF). Statistical analyses shown in Figs 2C and D, S2C–E, 5B, and S5B were determined by a paired *t* test, using GraphPad Prism 7 software. The statistical details of these experiments can be found in the figures and figure legends. Statistical analyses shown in Figs 1B and C, 3G, and 4C were determined by one-Way ANOVA and Dunnett’s multiple comparison test using GraphPad Prism 7 software. All experimental values are reported as the mean ± SD of three independent experiments.

### Data Availability

The Noc3 protein sequence was obtained from the Yeast Genome Database (https://www.yeastgenome.org/locus/S000003992/protein). DNA sequence homology was examined in terms of percentage, using the ClustalW2 online tool available from EMBL-EBI (http://www.ebi.ac.uk/). The coiled coil motifs were predicted by the COILS server with a 28-a.a. window (Lupas et al, 1991), while the MultiCoil program was used to predict the dimeric/trimeric coiled coil regions with a 28-a.a. window (Wolf et al, 1997) available from MIT CSAIL (https://cb.csail.mit.edu/cb/multicoil/cgi-bin/multicoil.cgi). This study did not generate any unique datasets or code. This study includes no data deposited in external repositories.

## Supplementary Material

Reviewer comments

## References

[bib1] Amin A, Cheung MH, Liang C (2019) DNA replication-initiation proteins in eukaryotic cells. Biomed J Sci Tech Res 23: 17042–17049. 10.26717/BJSTR.2019.23.003830

[bib2] Amin A, Wu R, Cheung MH, Scott JF, Wang Z, Zhou Z, Liu C, Zhu G, Wong CKC, Yu Z, (2020) An essential and cell cycle-dependent ORC dimerization cycle regulates eukaryotic chromosomal DNA replication. Cell Rep 30: 3323–3338.e6. 10.1016/j.celrep.2020.02.046 32160540

[bib3] Bassler J, Kallas M, Hurt E (2006) The NUG1 GTPase reveals an N-terminal RNA-binding domain that is essential for association with 60 S pre-ribosomal particles. J Biol Chem 281: 24737–24744. 10.1074/jbc.m604261200 16803892

[bib4] Bell SP, Labib K (2016) Chromosome duplication in *Saccharomyces cerevisiae*. Genetics 203: 1027–1067. 10.1534/genetics.115.186452 27384026 PMC4937469

[bib5] Ben-Aroya S, Coombes C, Kwok T, O’Donnell KA, Boeke JD, Hieter P (2008) Toward a comprehensive temperature-sensitive mutant repository of the essential genes of Saccharomyces cerevisiae. Mol Cell 30: 248–258. 10.1016/j.molcel.2008.02.021 18439903 PMC4130347

[bib6] Bueno A, Russell P (1992) Dual functions of CDC6: A yeast protein required for DNA replication also inhibits nuclear division. EMBO J 11: 2167–2176. 10.1002/j.1460-2075.1992.tb05276.x 1600944 PMC556684

[bib7] Calzada A, Sacristan M, Sanchez E, Bueno A (2001) Cdc6 cooperates with Sic1 and Hct1 to inactivate mitotic cyclin-dependent kinases. Nature 412: 355–358. 10.1038/35085610 11460169

[bib8] Chen S, de Vries MA, Bell SP (2007) Orc6 is required for dynamic recruitment of Cdt1 during repeated Mcm2-7 loading. Genes Dev 21: 2897–2907. 10.1101/gad.1596807 18006685 PMC2049192

[bib9] Chen VP, Choi RC, Chan WK, Leung KW, Guo AJ, Chan GK, Luk WK, Tsim KW (2011) The assembly of proline-rich membrane anchor (PRiMA)-linked acetylcholinesterase enzyme: Glycosylation is required for enzymatic activity but not for oligomerization. J Biol Chem 286: 32948–32961. 10.1074/jbc.m111.261248 21795704 PMC3190869

[bib10] Cheung MH, Amin A, Wu R, Qin Y, Zou L, Yu Z, Liang C (2019) Human NOC3 is essential for DNA replication licensing in human cells. Cell Cycle 18: 605–620. 10.1080/15384101.2019.1578522 30741601 PMC6464578

[bib11] Costa A, Hood IV, Berger JM (2013) Mechanisms for initiating cellular DNA replication. Annu Rev Biochem 82: 25–54. 10.1146/annurev-biochem-052610-094414 23746253 PMC4696014

[bib12] Coster G, Diffley JFX (2017) Bidirectional eukaryotic DNA replication is established by quasi-symmetrical helicase loading. Science 357: 314–318. 10.1126/science.aan0063 28729513 PMC5608077

[bib14] Diffley JFX (2011) Quality control in the initiation of eukaryotic DNA replication. Philos Trans R Soc B Biol Sci 366: 3545–3553. 10.1098/rstb.2011.0073 PMC3203456 22084381

[bib15] Dlakić M, Tollervey D (2004) The Noc proteins involved in ribosome synthesis and export contain divergent HEAT repeats. RNA 10: 351–354. 10.1261/rna.5184704 14970380 PMC1370930

[bib16] Dominguez C, Boelens R, Bonvin AMJJ (2003) HADDOCK: A protein-protein docking approach based on biochemical or biophysical information. J Am Chem Soc 125: 1731–1737. 10.1021/ja026939x 12580598

[bib17] Du YCN, Stillman B (2002) Yph1p, an ORC-interacting protein: Potential links between cell proliferation control, DNA replication, and ribosome biogenesis. Cell 109: 835–848. 10.1016/s0092-8674(02)00773-0 12110181

[bib18] Eaton ML, Galani K, Kang S, Bell SP, MacAlpine DM (2010) Conserved nucleosome positioning defines replication origins. Genes Dev 24: 748–753. 10.1101/gad.1913210 20351051 PMC2854390

[bib19] Evrin C, Clarke P, Zech J, Lurz R, Sun J, Uhle S, Li H, Stillman B, Speck C (2009) A double-hexameric MCM2-7 complex is loaded onto origin DNA during licensing of eukaryotic DNA replication. Proc Natl Acad Sci U S A 106: 20240–20245. 10.1073/pnas.0911500106 19910535 PMC2787165

[bib20] Fernández-Cid A, Riera A, Tognetti S, Herrera MC, Samel S, Evrin C, Winkler C, Gardenal E, Uhle S, Speck C (2013) An ORC/Cdc6/MCM2-7 complex is formed in a multistep reaction to serve as a platform for MCM double-hexamer assembly. Mol Cell 50: 577–588. 10.1016/j.molcel.2013.03.026 23603117

[bib21] Foss M, McNally FJ, Laurenson P, Rine J (1993) Origin recognition complex (ORC) in transcriptional silencing and DNA replication in S. cerevisiae. Science 262: 1838–1844. 10.1126/science.8266071 8266071

[bib22] Geisberg JV, Struhl K (2004) Quantitative sequential chromatin immunoprecipitation, a method for analyzing co-occupancy of proteins at genomic regions in vivo. Nucleic Acids Res 32: e151. 10.1093/nar/gnh148 15520460 PMC528824

[bib23] Georgescu R, Yuan Z, Bai L, de Luna Almeida Santos R, Sun J, Zhang D, Yurieva O, Li H, O’Donnell ME (2017) Structure of eukaryotic CMG helicase at a replication fork and implications to replisome architecture and origin initiation. Proc Natl Acad Sci U S A 114: E697–E706. 10.1073/pnas.1620500114 28096349 PMC5293012

[bib24] Gupta S, Friedman LJ, Gelles J, Bell SP (2021) A helicase-tethered ORC flip enables bidirectional helicase loading. Elife 10: e74282. 10.7554/elife.74282 34882090 PMC8828053

[bib25] Haruki H, Nishikawa J, Laemmli UK (2008) The anchor-away technique: Rapid, conditional establishment of yeast mutant phenotypes. Mol Cell 31: 925–932. 10.1016/j.molcel.2008.07.020 18922474

[bib26] Heller RC, Kang S, Lam WM, Chen S, Chan CS, Bell SP (2011) Eukaryotic origin-dependent DNA replication in vitro reveals sequential action of DDK and S-CDK kinases. Cell 146: 80–91. 10.1016/j.cell.2011.06.012 21729781 PMC3204357

[bib27] Honey S, Futcher B (2007) Roles of the CDK phosphorylation sites of yeast Cdc6 in chromatin binding and rereplication. Mol Biol Cell 18: 1324–1336. 10.1091/mbc.e06-06-0544 17267692 PMC1838967

[bib28] Huang S, Xu X, Wang G, Lu G, Xie W, Tao W, Zhang H, Jiang Q, Zhang C (2016a) DNA replication initiator Cdc6 also regulates ribosomal DNA transcription initiation. J Cell Sci 129: 1429–1440. 10.1242/jcs.178723 26872786

[bib29] Huang Y, Amin A, Qin Y, Wang Z, Jiang H, Liang L, Shi L, Liang C (2016b) A role of hIPI3 in DNA replication licensing in human cells. PLoS One 11: e0151803. 10.1371/journal.pone.0151803 27057756 PMC4825987

[bib30] Huo L, Wu R, Yu Z, Zhai Y, Yang X, Chan TC, Yeung JT, Kan J, Liang C (2012) The Rix1 (Ipi1p-2p-3p) complex is a critical determinant of DNA replication licensing independent of their roles in ribosome biogenesis. Cell Cycle 11: 1325–1339. 10.4161/cc.19709 22421151

[bib31] Johmura Y, Osada S, Nishizuka M, Imagawa M (2008a) FAD24 acts in concert with histone acetyltransferase HBO1 to promote adipogenesis by controlling DNA replication. J Biol Chem 283: 2265–2274. 10.1074/jbc.M707880200 18029353

[bib32] Johmura Y, Osada S, Nishizuka M, Imagawa M (2008b) FAD24, a regulator of adipogenesis, is required for the regulation of DNA replication in cell proliferation. Biol Pharm Bull 31: 1092–1095. 10.1248/bpb.31.1092 18520036

[bib33] Kan J, Zou L, Zhang J, Wu R, Wang Z, Liang C (2008) Origin recognition complex (ORC) mediates histone 3 lysine 4 methylation through cooperation with Spp1 in Saccharomyces cerevisiae. J Biol Chem 283: 33803–33807. 10.1074/jbc.C800182200 18845545 PMC2662210

[bib34] Kater L, Thoms M, Barrio-Garcia C, Cheng J, Ismail S, Ahmed YL, Bange G, Kressler D, Berninghausen O, Sinning I, (2017) Visualizing the assembly pathway of nucleolar pre-60S ribosomes. Cell 171: 1599–1610.e14. 10.1016/j.cell.2017.11.039 29245012 PMC5745149

[bib35] Kroll ES, Hyland KM, Hieter P, Li JJ (1996) Establishing genetic interactions by a synthetic dosage lethality phenotype. Genetics 143: 95–102. 10.1093/genetics/143.1.95 8722765 PMC1207298

[bib36] Kyei Barffour I, Acheampong DO (2021) Prospect of reprogramming replication licensing for cancer drug development. Biomed Pharmacother 136: 111190. 10.1016/j.biopha.2020.111190 33497909

[bib37] Lee DG, Bell SP (1997) Architecture of the yeast origin recognition complex bound to origins of DNA replication. Mol Cell Biol 17: 7159–7168. 10.1128/MCB.17.12.7159 9372948 PMC232573

[bib38] Lee C, Liachko I, Bouten R, Kelman Z, Tye BK (2010) Alternative mechanisms for coordinating polymerase α and MCM helicase. Mol Cell Biol 30: 423–435. 10.1128/MCB.01240-09 19917723 PMC2798462

[bib39] Lei M, Kawasaki Y, Tye BK (1996) Physical interactions among Mcm proteins and effects of Mcm dosage on DNA replication in Saccharomyces cerevisiae. Mol Cell Biol 16: 5081–5090. 10.1128/MCB.16.9.5081 8756666 PMC231509

[bib40] Li N, Zhai Y, Zhang Y, Li W, Yang M, Lei J, Tye BK, Gao N (2015) Structure of the eukaryotic MCM complex at 3.8 Å. Nature 524: 186–191. 10.1038/nature14685 26222030

[bib41] Li N, Lam WH, Zhai Y, Cheng J, Cheng E, Zhao Y, Gao N, Tye BK (2018) Structure of the origin recognition complex bound to DNA replication origin. Nature 559: 217–222. 10.1038/s41586-018-0293-x 29973722

[bib42] Liang C, Stillman B (1997) Persistent initiation of DNA replication and chromatin-bound MCM proteins during the cell cycle in cdc6 mutants. Genes Dev 11: 3375–3386. 10.1101/gad.11.24.3375 9407030 PMC316796

[bib43] Lin YC, Prasanth SG (2021) Replication initiation: Implications in genome integrity. DNA Repair 103: 103131. 10.1016/j.dnarep.2021.103131 33992866 PMC8296962

[bib44] Lipford JR, Bell SP (2001) Nucleosomes positioned by ORC facilitate the initiation of DNA replication. Mol Cell 7: 21–30. 10.1016/s1097-2765(01)00151-4 11172708

[bib45] Longtine MS, Mckenzie III A, Demarini DJ, Shah NG, Wach A, Brachat A, Philippsen P, Pringle JR (1998) Additional modules for versatile and economical PCR-based gene deletion and modification in Saccharomyces cerevisiae. Yeast 14: 953–961. 10.1002/(SICI)1097-0061(199807)14:10<953::AID-YEA293>3.0.CO;2-U 9717241

[bib46] Loo S, Fox CA, Rine J, Kobayashi R, Stillman B, Bell S (1995) The origin recognition complex in silencing, cell cycle progression, and DNA replication. Mol Biol Cell 6: 741–756. 10.1091/mbc.6.6.741 7579692 PMC301233

[bib47] Lupas A, Van Dyke M, Stock J (1991) Predicting coiled coils from protein sequences. Science 252: 1162–1164. 10.1126/science.252.5009.1162 2031185

[bib48] Ma LJ, Zhai YL, Feng DR, Chan TC, Lu Y, Fu XR, Wang JF, Chen YH, Li JN, Xu K, (2010) Identification of novel factors involved in or regulating initiation of DNA replication by a genome-wide phenotypic screen in *Saccharomyces cerevisiae*. Cell Cycle 9: 4399–4410. 10.4161/cc.9.21.13679 20980819

[bib49] Massari ME, Murre C (2000) Helix-loop-helix proteins: Regulators of transcription in eucaryotic organisms. Mol Cell Biol 20: 429–440. 10.1128/MCB.20.2.429-440.2000 10611221 PMC85097

[bib50] Milkereit P, Gadal O, Podtelejnikov A, Trumtel S, Gas N, Petfalski E, Tollervey D, Mann M, Hurt E, Tschochner H (2001) Maturation and intranuclear transport of pre-ribosomes requires Noc proteins. Cell 105: 499–509. 10.1016/s0092-8674(01)00358-0 11371346

[bib51] Miller TCR, Locke J, Greiwe JF, Diffley JFX, Costa A (2019) Mechanism of head-to-head MCM double-hexamer formation revealed by cryo-EM. Nature 575: 704–710. 10.1038/s41586-019-1768-0 31748745 PMC6887548

[bib52] Moyle-Heyrman G, Zaichuk T, Xi L, Zhang Q, Uhlenbeck OC, Holmgren R, Widom J, Wang JP (2013) Chemical map of Schizosaccharomyces pombe reveals species-specific features in nucleosome positioning. Proc Natl Acad Sci U S A 110: 20158–20163. 10.1073/pnas.1315809110 24277842 PMC3864286

[bib53] Remus D, Beuron F, Tolun G, Griffith JD, Morris EP, Diffley JF (2009) Concerted loading of Mcm2-7 double hexamers around DNA during DNA replication origin licensing. Cell 139: 719–730. 10.1016/j.cell.2009.10.015 19896182 PMC2804858

[bib54] Robinson KA, Lopes JM (2000) SURVEY AND SUMMARY: *Saccharomyces cerevisiae* basic helix-loop-helix proteins regulate diverse biological processes. Nucleic Acids Res 28: 1499–1505. 10.1093/nar/28.7.1499 10710415 PMC102793

[bib55] Rudra D, Warner JR (2004) What better measure than ribosome synthesis? Genes Dev 18: 2431–2436. 10.1101/gad.1256704 15489289

[bib56] Sheu YJ, Stillman B (2010) The Dbf4-Cdc7 kinase promotes S phase by alleviating an inhibitory activity in Mcm4. Nature 463: 113–117. 10.1038/nature08647 20054399 PMC2805463

[bib57] Sun J, Kawakami H, Zech J, Speck C, Stillman B, Li H (2012) Cdc6-induced conformational changes in ORC bound to origin DNA revealed by cryo-electron microscopy. Structure 20: 534–544. 10.1016/j.str.2012.01.011 22405012 PMC3299985

[bib58] Sun J, Evrin C, Samel SA, Fernández-Cid A, Riera A, Kawakami H, Stillman B, Speck C, Li H (2013) Cryo-EM structure of a helicase loading intermediate containing ORC-Cdc6-Cdt1-MCM2-7 bound to DNA. Nat Struct Mol Biol 20: 944–951. 10.1038/nsmb.2629 23851460 PMC3735830

[bib59] Sun J, Fernandez-Cid A, Riera A, Tognetti S, Yuan Z, Stillman B, Speck C, Li H (2014) Structural and mechanistic insights into Mcm2-7 double-hexamer assembly and function. Genes Dev 28: 2291–2303. 10.1101/gad.242313.114 25319829 PMC4201289

[bib60] Takara TJ, Bell SP (2011) Multiple Cdt1 molecules act at each origin to load replication-competent Mcm2-7 helicases. EMBO J 30: 4885–4896. 10.1038/emboj.2011.394 22045335 PMC3243627

[bib61] Tanaka S, Araki H (2010) Regulation of the initiation step of DNA replication by cyclin-dependent kinases. Chromosoma 119: 565–574. 10.1007/s00412-010-0291-8 20686781

[bib62] Tanaka T, Knapp D, Nasmyth K (1997) Loading of an MCM protein onto DNA replication origins is regulated by Cdc6p and CDKs. Cell 90: 649–660. 10.1016/s0092-8674(00)80526-7 9288745

[bib63] Ticau S, Friedman L, Ivica NA, Gelles J, Bell SP (2015) Single-molecule studies of origin licensing reveal mechanisms ensuring bidirectional helicase loading. Cell 161: 513–525. 10.1016/j.cell.2015.03.012 25892223 PMC4445235

[bib64] Tominaga K, Johmura Y, Nishizuka M, Imagawa M (2004) Fad24, a mammalian homolog of Noc3p, is a positive regulator in adipocyte differentiation. J Cell Sci 117: 6217–6226. 10.1242/jcs.01546 15564382

[bib65] Toulmay A, Schneiter R (2006) A two-step method for the introduction of single or multiple defined point mutations into the genome of Saccharomyces cerevisiae. Yeast 23: 825–831. 10.1002/yea.1397 16921548

[bib66] Tsai HJ, Baller JA, Liachko I, Koren A, Burrack LS, Hickman MA, Thevandavakkam MA, Rusche LN, Berman J (2014) Origin replication complex binding, nucleosome depletion patterns, and a primary sequence motif can predict origins of replication in a genome with epigenetic centromeres. mBio 5: e01703-14. 10.1128/mBio.01703-14 25182328 PMC4173791

[bib67] Wang J, Wu R, Lu Y, Liang C (2010) Ctf4p facilitates Mcm10p to promote DNA replication in budding yeast. Biochem Biophys Res Commun 395: 336–341. 10.1016/j.bbrc.2010.04.006 20381454

[bib68] Wang J, Amin A, Cheung MH, Shi L, Liang C (2022) Targeted inhibition of the expression of both MCM5 and MCM7 by miRNA-214 impedes DNA replication and tumorigenesis in hepatocellular carcinoma cells. Cancer Lett 539: 215677. 10.1016/j.canlet.2022.215677 35490917

[bib69] Warner MD, Azmi IF, Kang S, Zhao Y, Bell SP (2017) Replication origin-flanking roadblocks reveal origin-licensing dynamics and altered sequence dependence. J Biol Chem 292: 21417–21430. 10.1074/jbc.m117.815639 29074622 PMC5766963

[bib70] Weinreich M, Liang C, Stillman B (1999) The Cdc6p nucleotide-binding motif is required for loading mcm proteins onto chromatin. Proc Natl Acad Sci U S A 96: 441–446. 10.1073/pnas.96.2.441 9892652 PMC15155

[bib71] Weinreich M, Liang C, Chen HH, Stillman B (2001) Binding of cyclin-dependent kinases to ORC and Cdc6p regulates the chromosome replication cycle. Proc Natl Acad Sci U S A 98: 11211–11217. 10.1073/pnas.201387198 11572976 PMC58709

[bib72] Wolf E, Kim PS, Berger B (1997) MultiCoil: A program for predicting two-and three-stranded coiled coils. Protein Sci 6: 1179–1189. 10.1002/pro.5560060606 9194178 PMC2143730

[bib73] Wu R, Wang J, Liang C (2012) Cdt1p, through its interaction with Mcm6p, is required for the formation, nuclear accumulation and chromatin loading of the MCM complex. J Cell Sci 125: 209–219. 10.1242/jcs.094169 22250202

[bib74] Wu R, Amin A, Wang Z, Huang Y, Man-Hei Cheung M, Yu Z, Yang W, Liang C (2019) The Interaction networks of the budding yeast and human DNAreplication-initiation proteins. Cell Cycle 18: 723–741. 10.1080/15384101.2019.1586509 30890025 PMC6464591

[bib75] Yankulov K, Todorov I, Romanowski P, Licatalosi D, Cilli K, McCracken S, Laskey R, Bentley DL (1999) MCM proteins are associated with RNA polymerase II holoenzyme. Mol Cell Biol 19: 6154–6163. 10.1128/MCB.19.9.6154 10454562 PMC84545

[bib76] Yardimci H, Walter JC (2014) Prereplication-complex formation: A molecular double take? Nat Struct Mol Biol 21: 20–25. 10.1038/nsmb.2738 24389553

[bib77] Yeeles JTP, Deegan TD, Janska A, Early A, Diffley JFX (2015) Regulated eukaryotic DNA replication origin firing with purified proteins. Nature 519: 431–435. 10.1038/nature14285 25739503 PMC4874468

[bib78] Yuan Z, Riera A, Bai L, Sun J, Nandi S, Spanos C, Chen ZA, Barbon M, Rappsilber J, Stillman B, (2017) Structural basis of Mcm2-7 replicative helicase loading by ORC-Cdc6 and Cdt1. Nat Struct Mol Biol 24: 316–324. 10.1038/nsmb.3372 28191893 PMC5503505

[bib79] Zegerman P, Diffley JFX (2006) Phosphorylation of Sld2 and Sld3 by cyclin-dependent kinases promotes DNA replication in budding yeast. Nature 445: 281–285. 10.1038/nature05432 17167417

[bib80] Zhai Y, Yung PYK, Huo L, Liang C (2010) Cdc14p resets the competency of replication licensing by dephosphorylating multiple initiation proteins during mitotic exit in budding yeast. J Cell Sci 123: 3933–3943. 10.1242/jcs.075366 20980394

[bib81] Zhai Y, Yung PYK, Liang C (2011) Cell cycle control of DNA replication by phosphorylation and dephosphorylation of replication-initiation proteins in budding yeast. In Fundamental Aspects of DNA Replication, Kušić-Tišma J (ed), pp 87–106. London: InTech.

[bib82] Zhai Y, Cheng E, Wu H, Li N, Yung PYK, Gao N, Tye BK (2017) Open-ringed structure of the Cdt1-Mcm2-7 complex as a precursor of the MCM double-hexamer. Nat Struct Mol Biol 24: 300–308. 10.1038/nsmb.3374 28191894

[bib83] Zhang Y, Yu Z, Fu X, Liang C (2002) Noc3p, a bHLH protein, plays an integral role in the initiation of DNA replication in budding yeast. Cell 109: 849–860. 10.1016/s0092-8674(02)00805-x 12110182

[bib84] Zhou D, Zhu X, Zheng S, Tan D, Dong MQ, Ye K (2019) Cryo-EM structure of an early precursor of large ribosomal subunit reveals a half-assembled intermediate. Protein Cell 10: 120–130. 10.1007/s13238-018-0526-7 29557065 PMC6340896

