# Supplementary material for: An essential Noc3p dimerization cycle mediates ORC double-hexamer formation in replication licensing
Source: Life Sci Alliance. 2023 Jan 4;6(3):e202201594. doi: 10.26508/lsa.202201594 (PMC9813392; doi:10.26508/lsa.202201594)
Supplement: Supplementary file 1 [file LSA-2022-01594_TableS1.doc]

**Sup. Table S1. Key Resources Table**

| **Reagent/Resource** | **Source** | **Identifier** |
| --- | --- | --- |
| **Antibodies** | | |
| Anti-Orc3 (Mouse) | Gift from Prof. Bruce Stillman (CSH) | N/A |
| Anti-Orc6 (Mouse) | Gift from Prof. Bruce Stillman (CSH) | N/A |
| Anti-Mcm2 (Mouse) | Gift from Prof. Bruce Stillman (CSH) | N/A |
| Anti-Cdc6 (Goat) | Santa Cruz Biotechnology, Inc. | Cat#sc-6317; RRID:AB_638343 |
| Anti-HA (Mouse) | Roche Diagnostics | Cat#11666606001: RRID:AB_514506 |
| Anti-Myc (Mouse) | Roche Diagnostics | Cat#11667203001; RRID:AB_390911 |
| Anti-FLAG M2 (Mouse) | Sigma-Aldrich | Cat# F3165; RRID:AB_259529 |
| Anti-Histone H3 antibody (Rabbit) | Abcam | Cat#ab1791; RRID:AB_302613 |
| Anti-beta Actin antibody - Loading Control | Abcam | Cat#ab8226; RRID:AB_306371 |
| Donkey anti-Goat IgG (H+L) Secondary Antibody, HRP | ThermoFisher Scientific, Inc. | Cat#A15999; RRID:AB_2763439 |
| Goat anti-Rabbit IgG (H+L) Highly Cross-Adsorbed Secondary Antibody, HRP | ThermoFisher Scientific, Inc. | Cat#A16110; RRID:AB_2534782 |
| Goat anti-Mouse IgG (H+L) Secondary Antibody, HRP | ThermoFisher Scientific, Inc. | Cat#31430; RRID:AB_228307 |
| Peroxidase IgG Fraction Monoclonal Mouse Anti-Rabbit IgG light chain specific | Jackson ImmunoResearch Laboratories, Inc. | Cat#211-032-171; RRID:AB_2339149 |
| Peroxidase AffiniPure Goat Anti-Mouse IgG light chain specific | Jackson ImmunoResearch Laboratories, Inc. | Cat#115-035-174; RRID:AB_2338512 |
| **Bacterial Strains** | | |
| Subcloning Efficiency™ DH5α Competent Cells | ThermoFisher Scientific, Inc. | Cat# 18265017 |
| **Chemicals, Peptides, and Recombinant Proteins** | | |
| Hydroxyurea | Sigma-Aldrich | Cat# H8627 |
| Nocodozole | Sigma-Aldrich | Cat# M1404-50MG |
| Alpha-Factor | Sangon Biotech (Shanghai) Co., Ltd. | Custom Synthesis |
| SuperSignal West Pico PLUS | ThermoFisher Scientific, Inc. | Cat# 34580 |
| Rec-Protein G - Sepharose 4B Conjugate | Invitrogen | Cat# 101243 |
| Formaldehyde solution | Sigma-Aldrich | Cat# 252549-2.5L |
| Glycine Ultrapure MB Grade | Affymetrix, Inc. | Cat# 16407 5 KG |
| BioRad - Protein Assay Dye Reagent Concentrate | Bio-Rad Laboratories, Inc. | Cat# 500-0006 |
| DNase I recombinant, RNase-free | Roche Diagnostics | Cat# 04716728001 |
| Yeast Lytic Enzyme | MP Biomedicals | Cat# 02153526-CF |
| Alkaline Phosphatase | New England BioLabs, Inc. | Cat# M0290 |
| Beta-galactosidase | Roche Diagnostics | Cat# 10105031001 |
| **Experimental Models: Organisms/Strains** | | |
| Saccharomyces cerevisiae. | NCBI | NCBI:txid4896 |
| W303-1A: MATalpha ade2-1 ura3-1 his3-11 trp1-1 leu2-3 leu2-112 can1-100 | ATCC | ATCC: 208353 |
| AH109: MATa, trp1-901, leu2-3, 112, ura3-52, his3-200, gal4Δ,gal80Δ, LYS2 : : GAL1UAS-GAL1TATA-HIS3, GAL2UAS-GAL2TATA-ADE2, URA3 : : MEL1UAS-MEL1 TATA-lacZ | Clontech | N/A |
| HHY212: MatA tor1-1 fpr::loxP-LEU-LoxP RPL13A-FK::LoxP-TRP-LoxP | (Haruki et al., 2008) ^66^ | N/A |
| noc3-1 | (Milkereit et al., 2001) ^22^ | N/A |
| noc3-ts-URA | (Ben-Aroya et al., 2008) ^60^ | N/A |
| NOC3-HA | (Zhang et al., 2002) ^9^ | N/A |
| See Table S2: full strain list. | This paper | N/A |
| **Recombinant DNA** | | |
| pGADT7 2μ, LEU2, Amp, GAL4-AD | Clontech | Cat# 630442 |
| pGBKT7 2μ, TRP1, Kan, GAL4-BD | Clontech | Cat# 630489 |
| pL1587: pRS316-zz-noc3-1 | (Milkereit et al., 2001) ^22^ | N/A |
| pL1596: pRS316-Rpl25-eGFP | (Milkereit et al., 2001) ^22^ | N/A |
| See Table S3: full plasmid list. | This paper | N/A |
| **Oligonucleotides** | | |
| GAL-Noc3 F: TGCCCGAAAACTGAAAAT  TTTATCTCATCTCATTCAC  CTGCCTGCCATATGAATT  CGAGCTCGTTTAAAC | This paper; Synthesized by Sangon Biotech (Shanghai) Co., Ltd. | N/A |
| GAL-Noc3 R:  TTCTTTGCAGTCCTTTCCT  GAATGCGAAATTGAGATC  TATTTCTCTTAGCGCACT  GAGCAGCGTAATCTG | This paper; Synthesized by Sangon Biotech (Shanghai) Co., Ltd. | N/A |
| Noc3 del F:  CATAAGAGTATAATTCCG  GGATAAAAGCTGGAGAT  ATCATAGTAATAATGCGT  ACGCTGCAGGTCGAC | This paper; Synthesized by Sangon Biotech (Shanghai) Co., Ltd. | N/A |
| Noc3 del R:  GCTAACGATAATCGTGGC  TCTTTATATACTTAATATA  TAGGATCTAGCTAATCGA  TGAATTCGAGCTCG | This paper; Synthesized by Sangon Biotech (Shanghai) Co., Ltd. | N/A |
| pRS414 F:  TGAGCGCGCGTAATACG  ACTC | This paper; Synthesized by Sangon Biotech (Shanghai) Co., Ltd. | N/A |
| pRS414 R:  GCTTCCGGCTCCTATGTT  TGTG | This paper; Synthesized by Sangon Biotech (Shanghai) Co., Ltd. | N/A |
| Noc3 S2:  TAATCGTGGCTCTTTATA  TACTTAATATATAGGATC  TAGTTAATCGATGAATTC  GAGCTCTTCGAGCTC | This paper; Synthesized by Sangon Biotech (Shanghai) Co., Ltd. | N/A |
| noc3-3 S3:  GAAATTTCAGGCCTTTAC  TCTTCCGAGGATAGAATA  GGCAACCGTACGCTGCA  GGTCGAC | This paper; Synthesized by Sangon Biotech (Shanghai) Co., Ltd. | N/A |
| Noc3S3:  TTACATGACTATGACCC  TAATACTAAATTGAAAG  GTAACGTTCGTACGCTG  CAGGTCGAC | This paper; Synthesized by Sangon Biotech (Shanghai) Co., Ltd. | N/A |
| **Software** | | |
| ImageJ (FIJI) | NIH | RRID:SCR_002285 |
| Microsoft Excel | Microsoft | RRID:SCR_016137 |
| Photoshop | Adobe | RRID:SCR_014199 |
| Graphpad Prism 7 software | Graphpad | RRID:SCR_002798 |
