# Supplementary material for: An essential Noc3p dimerization cycle mediates ORC double-hexamer formation in replication licensing
Source: Life Sci Alliance. 2023 Jan 4;6(3):e202201594. doi: 10.26508/lsa.202201594 (PMC9813392; doi:10.26508/lsa.202201594)
Supplement: Supplementary file 2 [file LSA-2022-01594_TableS2.doc]

**Sup. Table S2. Yeast Strains, Related to Methods**

| **Strain** | **Genotype** | **Source** |
| --- | --- | --- |
| YL768 | W303-1a *noc3Δ::noc3-3* | This paper |
| YL1923 | W303-1A, *cdc6 Δ::hisG,trp::Met3-CDC6::TRP* | This paper |
| YL1885 | HHY212, *ORC1-FRB-FLAG::natNT* | This paper |
| YLN3FRB | HHY212, *NOC3-FRB-FLAG::natNT* | This paper |
| YL816 | W303-1a *NOC3-6HA(HIS3)* | This paper |
| YL774 | W303-1a *noc3Δ::GAL-NOC3::KanMX* | This paper |
| YL1289 | W303-1a *orc5Δ::orc5-1* | This paper |
| YL1292 | W303-1a *mcm5Δ::mcm5-1* | This paper |
| YLnoc3-9 | W303-1a *noc3Δ::GAL- NOC3::KanMX noc3-9* | This paper |
| YLnoc3-142 | W303-1a *noc3Δ::GAL- NOC3::KanMX noc3-142* | This paper |
| YLnoc3-CC1Δ | W303-1a *noc3Δ::GAL- NOC3::KanMX Noc3-CC1Δ* | This paper |
| YLnoc3-CC2Δ | W303-1a *noc3Δ::GAL- NOC3::KanMX Noc3-CC2Δ* | This paper |
