# Supplementary material for: An essential Noc3p dimerization cycle mediates ORC double-hexamer formation in replication licensing
Source: Life Sci Alliance. 2023 Jan 4;6(3):e202201594. doi: 10.26508/lsa.202201594 (PMC9813392; doi:10.26508/lsa.202201594)
Supplement: Supplementary file 3 [file LSA-2022-01594_TableS3.doc]

**Sup. Table S3. Plasmids for Yeast Cell Transformation, Related to Methods**

| **Name** | **Genotype** | **Source** |
| --- | --- | --- |
| pL1016 | pGADT7-ORC2 | This paper |
| pL1018 | pGADT7-ORC3 | This paper |
| pL985 | pGADT7-NOC3 | This paper |
| pL1049 | pGBKT7-ORC2 | This paper |
| pL1052 | pGBKT7-ORC3 | This paper |
| pL991 | pGBKT7-NOC3 | This paper |
| pL1174 | pGBKT7-NOC3 F1 (a.a. 1-325) | This paper |
| pL1146 | pGBKT7-NOC3 F2 (a.a. 1-512) | This paper |
| pL1149 | pGBKT7-NOC3 F3 (a.a. 170-664) | This paper |
| pL1152 | pGBKT7-NOC3 F4 (a.a. 326-664) | This paper |
| pL989 | pGBKT7-NOC3 F5 (a.a. 1-169) | This paper |
| pL1072 | pGBKT7-NOC3 F6 (a.a. 170-325) | This paper |
| pL1074 | pGBKT7-NOC3 F7 (a.a. 326-512) | This paper |
| pL990 | pGBKT7-NOC3 F8 (a.a. 513-664) | This paper |
| AD-ΔN1 | pGADT7-ΔN1 | This paper |
| AD-ΔN2 | pGADT7-ΔN2 | This paper |
| AD-ΔCC1 | pGADT7-ΔCC1 | This paper |
| AD-ΔCC2 | pGADT7-ΔCC2 | This paper |
| BD-nocΔ | pGBKT7-nocΔ | This paper |
| BD-ΔCC1 | pGBKT7-ΔCC1 | This paper |
| pL1985 | pGBKT7-ΔCC2 | This paper |
| BD-D1 | pGBKT7-D1 | This paper |
| BD-D2 | pGBKT7-D2 | This paper |
| BD-D3 | pGBKT7-D3 | This paper |
| BD-D4 | pGBKT7-D4 | This paper |
| BD-nocΔ | pGBKT7-nocΔ | This paper |
| p1ARS | ARS1, CEN3, LEU2, ade3-2 | This paper |
| p8ARSs | ARS1, CEN3, LEU2, ade3-2, 7×H4ARS | This paper |
| pL1162 | pRS425 | This paper |
| pLpRS425-noc3 | pRS425-noc3 | This paper |
| pL1180 | pRS425-noc3-CC1Δ | This paper |
| pL1181 | pRS425-noc3-CC2Δ | This paper |
| pRS425-nocΔ | pRS425-nocΔ | This paper |
| pL596 | pRS416 | This paper |
| pRS416-N3 | pRS416-NOC3 | This paper |
| pL941 | pRS414 | This paper |
| pL1990 | pRS414-noc3-9 | This paper |
| pL1991 | pRS414-noc3-142 | This paper |
| pL1587 | pRS414-noc3-5 | This paper |
| pLnoc3ΔCT | pRS414-noc3ΔCT | This paper |
| pL1705 | pESC-Myc-ORC6/ORC6-FLAG | This paper |
| pL1967 | pESC-Myc-ORC2/ORC2-FLAG | This paper |
| pESC-N3X2 | pESC-Myc-NOC3/NOC3-FLAG | This paper |
| pESC-Myc-Noc3 | pESC-Myc-NOC3 | This paper |
| pESC-CC2ΔX2 | pESC-Myc-CC2Δ/CC2Δ-FLAG | This paper |
| pESC-CC2Δ-FLAG | pESC-CC2Δ-FLAG | This paper |
| pRS416-NOC3 | pRS416-NOC3 | This paper |
| pLpRS425-noc3-1 | pRS425-noc3-1 | This paper |
| pLpRS425-noc3-3 | pRS425-noc3-3 | This paper |
| pL1992 | pGAL-FLAG-ORC1 | This paper |
| pL1993 | pGAL-FLAG-ORC2 | This paper |
| pL1994 | pGAL-FLAG-ORC3 | This paper |
| pL1995 | pGAL-FLAG-ORC4 | This paper |
| pL1996 | pGAL-FLAG-ORC5 | This paper |
| pL1997 | pGAL-FLAG-ORC6 | This paper |
| pL1998 | pGAL-FLAG-MCM2 | This paper |
| pL1999 | pGAL-FLAG-MCM3 | This paper |
| pL2000 | pGAL-FLAG-MCM4 | This paper |
| pL2001 | pGAL-FLAG-MCM5 | This paper |
| pL2002 | pGAL-FLAG-MCM6 | This paper |
| pL2003 | pGAL-FLAG-MCM7 | This paper |
| pL2004 | pGAL-FLAG-CDT1 | This paper |
| pL2005 | pGAL-FLAG-CDC45 | This paper |
| pL2006 | pGAL-FLAG-SLD2 | This paper |
| pL2007 | pGAL-FLAG-SLD4 | This paper |
| pL2008 | pGAL-FLAG-DBP11 | This paper |
